# *Arabidopsis thaliana* Zn transporter genes *ZIP3* and *ZIP5* provide the main Zn uptake route and act redundantly to face Zn deficiency

**DOI:** 10.1111/tpj.17251

**Published:** 2025-02-10

**Authors:** Valeria Ochoa Tufiño, Maria Almira Casellas, Aron van Duynhoven, Paulina Flis, David E. Salt, Henk Schat, Mark G. M. Aarts

**Affiliations:** ^1^ Laboratory of Genetics Wageningen University 6700 AA Wageningen The Netherlands; ^2^ Future Food Beacon of Excellence & School of Biosciences University of Nottingham LE12 5RD Sutton Bonington UK; ^3^ Present address: Departmento de Ciencias de la Vida Universidad de las Fuerzas Armadas – ESPE Sangolquí Ecuador; ^4^ Present address: Universitat Autònoma de Barcelona 08193 Bellaterra Spain

**Keywords:** mineral homeostasis, nutrient deficiency, promoter‐YFP fusion, tissue‐specific gene expression, ZIP, Zinpyr‐1, Zn transporters

## Abstract

In response to Zn deficiency, plants are thought to adjust Zn homeostasis through the coordinated expression of Zn transporters. Zn transporters are identified in the ZIP, HMA and CDF families of cation transporters, although only few are characterized. We determined gene expression over time, root‐specific location of expression and phenotypes of single and double loss‐of‐function mutants of several *Arabidopsis thaliana* transporters, known to be induced by Zn deficiency. Transcription of Zn transporter genes is induced in the first 6 h of exposure to Zn deficiency. *ZIP1* is predominantly expressed in the endodermis and stele, *ZIP3* and *ZIP5* in the epidermis and cortex, *IRT3* from epidermis to stele and *HMA2* in xylem parenchyma. *ZIP3* and *ZIP5* act redundantly, with the double‐mutant *zip3zip5* showing high sensitivity to Zn deficiency with low biomass production, expression of other transporter genes, low Zn uptake and increased metal translocation. The root expression map and timing indicate that Zn transporters act complementary in a concerted action to control Zn homeostasis. The lack of strong Zn‐deficient phenotypes in single mutants suggests a high level of functional redundancy, best illustrated for *ZIP3* and *ZIP5*.

## Significance Statement

This is the first report on the root tissue‐specific expression of several Zn transporters in *Arabidopsis thaliana*, which illustrates how complementary they are expressed. It highlights ZIP3 and ZIP5 as two major ZIP transporters, expressed in the epidermis and cortex of the root and acting redundantly, which provide an essential function to take up sufficient Zn under Zn‐deficient conditions.

## INTRODUCTION

Zinc is an essential micronutrient for life, being the second most abundant transition element in all organisms, including plants (Broadley et al., 2007). Zinc deficiency in plants causes symptoms such as stunted growth, chlorosis and reduced yield (Alloway, 2009; Cakmak, 2008; Marschner & Marschner, 2012; Mattiello et al., 2015; Yruela, 2013). Plants are able to sense the shortage of Zn supply and adjust their internal Zn status through Zn homeostasis. The transcription factors bZIP19 and bZIP23 act as essential sensors for plant Zn status (Lilay et al., 2021). They control the Zn deficiency response through expression of a range of Zn transporter genes, thus increasing the Zn^2+^ uptake capacity to overcome Zn deficiency (Assunção et al., 2010; Assunção et al., 2013; Lilay et al., 2020; Lilay et al., 2021). Most of the known Zn transporters belong to the ZRT‐IRT‐like protein (ZIP), heavy‐metal ATPase (HMA) and cation diffusion facilitator (CDF) families (Eide, 2006; Eren & Argüello, 2004; Kobae et al., 2004; Sinclair & Krämer, 2012). ZIPs transport divalent cations from the extracellular space or organelle lumen into the symplast (Eide, 2006). *Arabidopsis thaliana* has 15 *ZIP* genes (Guerinot, 2000; Milner et al., 2013). Evidence that associates members of the *A. thaliana ZIP* family with Zn transport is provided through differential gene expression, yeast functional complementation and sequence similarity (Grotz et al., 1998; Milner et al., 2013; van de Mortel et al., 2006). Zn deficiency induces expression of *ZIP1*, *ZIP3*, *ZIP4*, *ZIP5*, *ZIP9*, *ZIP10*, *ZIP12* and *IRT3* (Assunção et al., 2010; Campos, 2015; van de Mortel et al., 2006). The *ZIP1, ZIP2, ZIP3, ZIP4, IRT1* and *IRT3* coding sequences complement the *zrt1zrt2* yeast mutant defective in Zn uptake, consistently among studies (Assunção et al., 2010; Grotz et al., 1998; Korshunova et al., 1999; Lee et al., 2021; Lin et al., 2009; Milner et al., 2013; Vert et al., 2001). These results strongly support an important role of especially ZIP1, ZIP3, ZIP4 and IRT3, to rapidly supply the plant with extra Zn uptake capacity upon Zn deficiency. Most of the ZIP transporters must act redundantly, as single knock‐out mutants often do not display an aberrant phenotype, or only a weak one (Inaba et al., 2015; Lee et al., 2021; Milner et al., 2013; Wu et al., 2009). While many of the *ZIP* genes are known for decades, this redundancy has not yet been resolved.

The CDF and HMA proteins transport cations from the symplast to outside the cell or into subcellular compartments. In plants, CDFs are usually called Metal Tolerance Proteins (MTPs) (Montanini et al., 2007; Paulsen & Saier Jr, 1997). The *A. thaliana* MTP1 and MTP3 are tonoplast‐localized and mediate the Zn efflux from the symplast to the vacuole (Arrivault et al., 2006; Desbrosses‐Fonrouge et al., 2005; Kobae et al., 2004). *MTP2* (Dräger et al., 2004) is the only *MTP* induced by Zn deficiency (van de Mortel et al., 2006). MTP2 restores Zn tolerance in a hypersensitive yeast mutant (Sinclair et al., 2018) and acts additively with HEAVY METAL ATPASE 2 (HMA2) in the partitioning of Zn from roots to shoots. HMA2 ATPase activity is activated by Zn (Eren & Argüello, 2004) and *HMA2* expression is induced by Zn deficiency (van de Mortel et al., 2006). *HMA2* and *HMA4* are expressed in the vasculature of roots, stems and leaves, and they act redundantly to promote Zn translocation from roots to shoots (Hussain et al., 2004; Sinclair et al., 2007).

The Zn supply to *A. thaliana* shoots depends on how effectively roots acquire Zn and make it available for translocation to shoots. In general, nutrients move radially through the root, via epidermis, cortex and endodermis, before entering the stele, where nutrients can be loaded into the vasculature. This transport can be apoplastic, that is by passive transport through cell walls and intercellular spaces, or symplastic, mediated by influx and efflux carriers (transporters) and from cell‐to‐cell through plasmodesmata, or in a combination of both, the coupled pathway (Barberon & Geldner, 2014; Clarkson, 1993; Geldner, 2013; Tester & Leigh, 2001). The endodermis is a crucial checkpoint for the control on nutrient intake, as apoplastic transport beyond the endodermis is prevented due to the presence of Casparian Strips, tangential depositions of lignin around the endodermal cells and suberin lamellae deposited at a slightly later stage around the endodermis, which block virtually all apoplastic transport (Barberon, 2017). At later stages of root development, transport of nutrients to the stele can only occur through the so‐called passage cells, which are free from suberin lamellae (Ramakrishna & Barberon, 2019).

So far, surprisingly little is known on Zn transport in roots, even though several of the Zn transporters are known. This is probably due to the genetic redundancy of the transporter genes. With the aim of filling this knowledge gap, we follow Zn fluxes using Zinpyr‐1 in the roots of wild‐type plants and loss‐of‐function mutants of several well‐known Zn transporters. Based on promoter and gene expression studies, we identify the root cell layer(s) where each transporter is expressed and how their expression levels change over time. Moreover, we determine the ionome effects of single and double loss‐of‐function Zn transporter mutants, and corroborate the expected redundancy and functional overlap of *A. thaliana* Zn transporters.

## RESULTS

### Zn deficiency response is detected after 6 h

**Figure 1:**
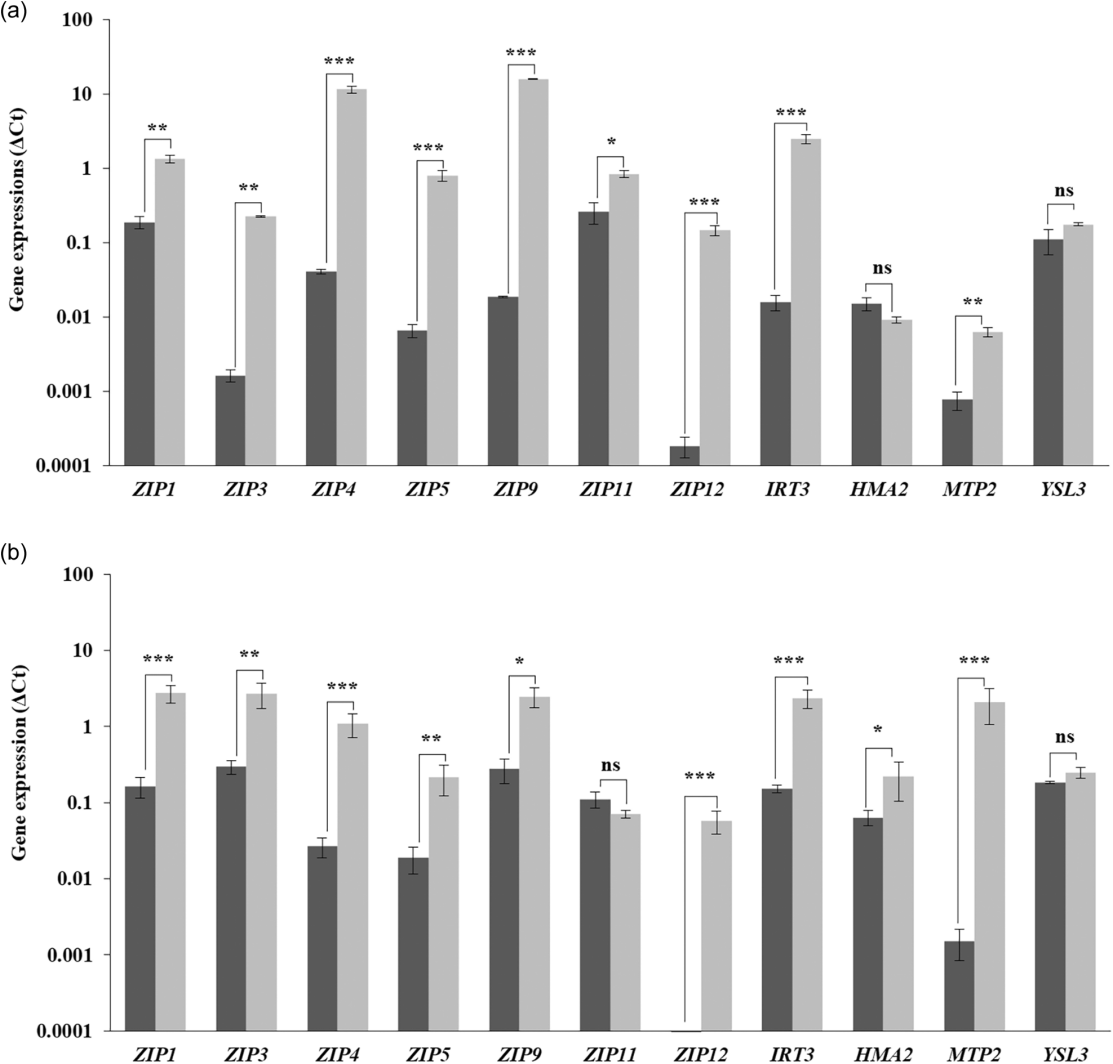
Normalized gene expressions of Zn transporter genes in shoots (a) and roots (b) of *A. thaliana* Col‐0 plants exposed to Zn sufficient (dark bar) and Zn deficient hydroponic medium (light bar). 15‐day‐old plants grown on Zn sufficient medium are transferred to either fresh Zn sufficient medium or Zn deficient medium, and sampled 12.5 days after transfer. The Zn transporter genes tested belong to the ZRT‐IRT‐like protein (ZIP), heavy‐metal ATPase (HMA), Metal Tolerance Protein (MTP), and Yellow Stripe1‐Like (YSL) gene families. Gene expression is normalized to the expression of two reference genes (At5g25760 and AT2G28390). Average normalized expressions ± SE are shown on a ^10^log‐scale, *n* = 3 samples of 3 pooled plants each. Statistical significance for gene expression difference is determined by Student's *t*‐test (*P < 0.05, **P < 0.01, and ***P < 0.001).

We determined the induction of Zn transporter gene expression upon Zn deficiency in order to establish the coordination of expression in time and place. At 6 h of treatment, the first significant increase in expression of Zn transporter genes in response to Zn deficiency is detected for *ZIP1* and *ZIP4*, in roots (Table 1 and Figure S1). At 12‐h treatment, *ZIP3*, *ZIP5* and *IRT3* are significantly induced in roots and *IRT3* also in shoots. Next to the short‐term responses, determined over a period of 4.5 days (108 h), we also determined gene expression in shoots and roots after 12.5 days (300 h), when plants have initiated the Zn deficiency response in full (Figure 1). After 12.5 days, most of the tested genes in shoots (Figure 1a), and in roots (Figure 1b) are (much) higher expressed in response to Zn deficiency when compared to plants grown in Zn sufficiency. The highest expressed genes upon Zn deficiency treatment after 12.5 days of exposure to Zn deficiency are *ZIP4* and *ZIP9* in shoots and *ZIP1, ZIP3, ZIP9, IRT3* and *MTP2* in roots (Figure 1). *ZIP9*, together with *ZIP12*, is also most induced in shoots by Zn deficiency after 12.5 days, when compared to Zn sufficiency, while *ZIP12* and *MTP2* are most induced in roots. Please note, the fold‐change induction of *ZIP12* expression is very high as expression of this gene is barely detectable at Zn sufficiency.

**Table 1:** Fold‐changes of normalized Zn transporter gene expression over time, in shoot and roots of *A. thaliana* plants exposed to Zn deficiency compared to controls growing in Zn sufficient hydroponic medium

	*ZIP1*	*ZIP3*	*ZIP4*	*ZIP5*	*ZIP9*	*ZIP11*	*ZIP12*	*IRT3*	*HMA2*	*MTP2*	*YSL3*
Shoot											
0.25 h	0.9	0.8	0.8	0.6	0.9	0.8	0.6	0.7	1.0	0.5	0.8
1 h	0.7	0.9	1.4	0.4	0.7	0.8	0.1	0.7	0.6	0.8	0.8
3 h	1.0	0.7	1.1	0.7	0.9	1.0	0.6	1.2	0.8	1.3	0.9
6 h	1.4	1.4	1.5	1.3	0.7	0.9	0.9	1.8	2.1	0.6	1.1
12 h	1.5	1.2	1.3	1.4	1.5	1.1	3.0	2.5*	1.2	1.1	1.3
36 h	0.4	0.8	0.8	0.7	0.5	0.4	0.5	1.0	0.2	0.7	1.2
108 h	2.0*	0.7	1.4*	2.3***	1.4	1.4	5.8*	4.5***	1.7**	2.2**	2.8**
Root											
0.25 h	1.1	0.7	0.9	0.8	0.8	2.3	0.3	0.6	1.2	0.3	0.6
1 h	2.0	0.9	1.5	0.5	1.2	2.3	3.8	1.1	0.6	0.4	0.2
3 h	1.9	1.1	2.4	1.3	0.9	1.1	1.4	1.7	0.7	0.2	1.2
6 h	3.3*	1.6	3.1**	1.6	1.0	1.7	0.9	1.9	0.7	1.2	0.4
12 h	2.7	2.1*	3.5	3.1*	0.9	1.6	4.4	5.9*	1.4	0.8	1.8
36 h	2.5*	1.8**	1.5	1.5	0.6	0.7	0.7	2.3*	0.9	0.6	2.8
108 h	3.4*	1.7	2.2*	1.4	1.3	1.2	0.4	2.4	0.4	2.1	5.0*

Plants are grown on Zn sufficient medium for 15 days, before transfer to Zn deficient medium. Samples are taken at the indicated time points after transfer (at 0.25, 1, 3, 6, 12, 36, and 108 h). Gene expressions are normalized to the average expression of two reference genes (At5g25760 and At2g28390) and compared to the normalized expression of the same genes in control plants, which are maintained in Zn sufficient medium, to calculate the average fold changes of expression at Zn deficiency compared to expression at Zn sufficiency. Statistical significance for gene expression differences between Zn deficiency and Zn sufficiency grown plants at each time point is determined by Student's *t* test (*P < 0.05, **P < 0.01, and ***P < 0.001). *n* = 3 samples of 3 pooled plants each.

HMA, heavy‐metal ATPase; MTP, Metal Tolerance Protein; YSL, Yellow Stripe1‐Like; ZIP, ZRT‐IRT‐like protein.

Not all of the genes we examined are significantly induced by Zn deficiency after 12.5 days of treatment. *HMA2* in shoots and *YSL3* in shoots and roots are only induced after 4.5 days of exposure to Zn deficiency (Table 1 and Figure S1). The expression of *ZIP11* is not affected by Zn deficiency in roots at any of the analysed time points. The expression levels of Zn transporter genes change over time. Both in roots and shoots, most of the genes follow a pattern of a weak initial induction of gene expression at 12 h, a drop in expression after 36 h, followed by a second peak in gene expression at 4.5 days, continuing to the often strong increase in expression visible after 12.5 days (Figure 1).

### Zn distribution in roots is altered in Zn transporter loss‐of‐function mutants

**Figure 2:**
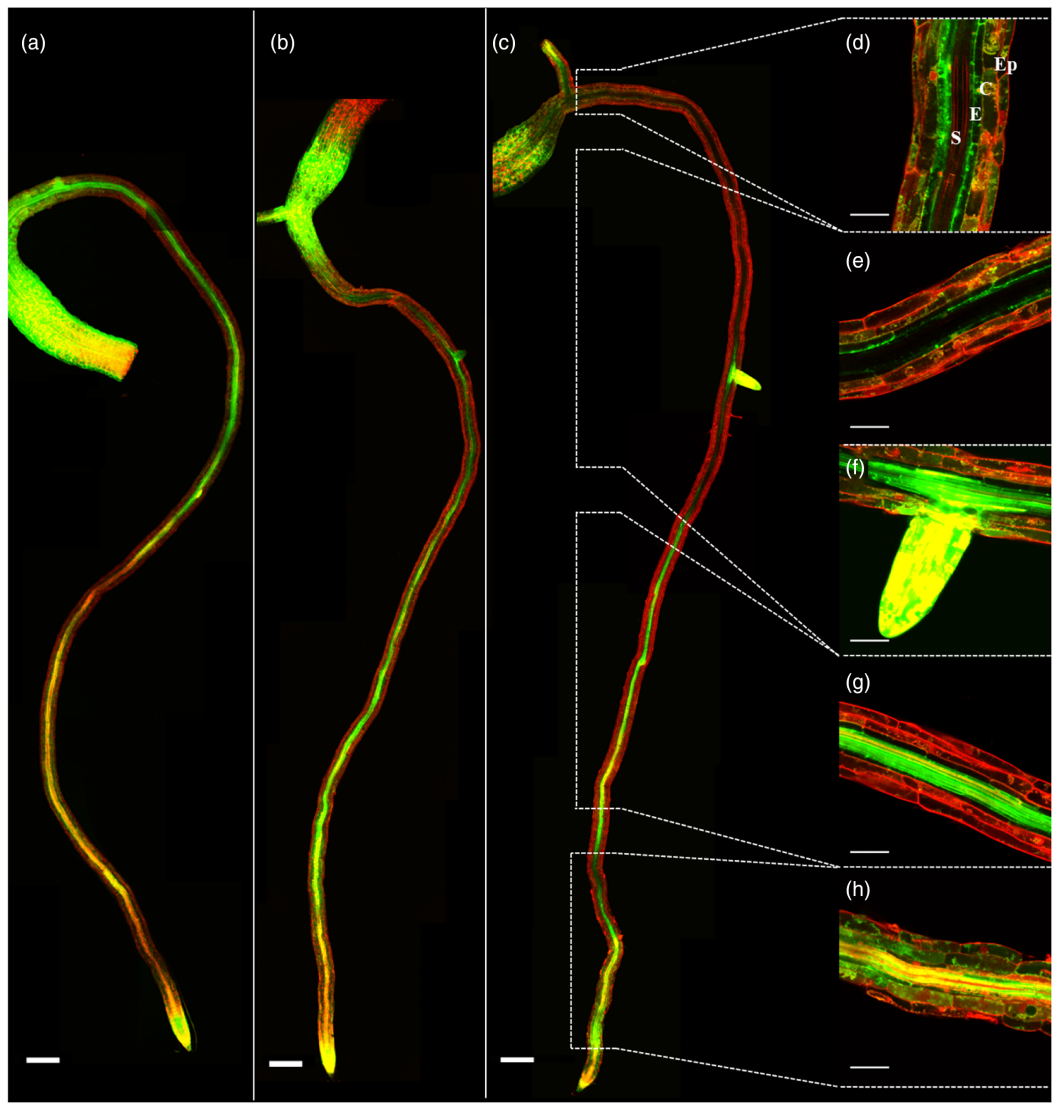
Confocal laser‐scanning microscope images of Zn distribution in *A. thaliana* wild‐type seedling roots. Roots of Zn deficient Col‐0 seedlings are incubated with Zinpyr‐1 to detect Zn (a) in five‐, (b) six‐ or (c) seven‐day‐old plants covering part of the hypocotyl and the root highlighting (d) the transition zone and the (h) root elongation transition zone. Zinpyr‐1‐Zn complexes fluoresce green; propidium iodide, which is mainly bound to cell walls, fluoresces red. Root cell layers are distinguished from the outside to inside as epidermis (Ep), cortex (Co), endodermis (En) and stele (St) as indicated in (d). The scale bars indicate 200 μm in (a)–(c), and 50 μm in (d)–(h).

In order to investigate how Zn accumulates in *A. thaliana* roots, we used the Zn fluorescent dye Zinpyr‐1 to visualize the distribution of free Zn^2+^ in roots of young (5–7 days old) *A. thaliana* wild‐type Col‐0 seedlings. Zinpyr‐1 is a fluorescein‐based bright green fluorescent Zn^2+^ sensor (Woodroofe et al., 2004). Its fluorescence intensity indicates relative levels of Zn, but as it is very sensitive, reliable relative concentration estimates can only be made at very low Zn^2+^ concentrations (Sinclair et al., 2007). This meant we only consider obvious differences in fluorescence to provide a relevant indication of different Zn concentrations under very low Zn supply and when observed in multiple samples. We could not apply this method for plants in Zn sufficiency conditions. Zn‐sufficient plants show strong fluorescence in all root layers, which does not allow for any differentiation of Zn distribution patterns (data not shown). When seedlings are grown in strong Zn deficiency inducing conditions (germination and growth in the absence of added Zn), such patterns are visible and will reflect how the plants prioritize their Zn distribution. The presence of Zn is examined in the whole root, from the root tip to the root–shoot transition region of the primary root (Figure 2). The root tip, lateral root primordia, lateral root tips and the root–shoot transition region show the highest intensity of the Zinpyr‐1 fluorescence, while the elongation zone shows the lowest intensity. In the differentiation zone, the Zinpyr‐1 fluorescence is most intense in the stele.

The Zinpyr‐1 fluorescence in the stele of five‐day‐old seedlings is more intense closer to the root‐shoot transition region (Figure 2a). In six‐day‐old seedlings, fluorescence in the stele close to the transition zone (Figure 2b) is less compared to five‐day‐old seedlings. In seven‐day‐old seedlings, the fluorescence is almost absent in the stele in the older parts of the root (Figure 2c). In these parts, the Zinpyr‐1 fluorescence is found along the epidermis, cortex and endodermis (Figure 2d,e). In five‐, six‐ and seven‐day‐old seedlings, the Zinpyr‐1 fluorescence is consistently high in root zones with dividing cells, suggesting that the remaining Zn is (re)mobilized to growing tissues.

**Figure 3:**
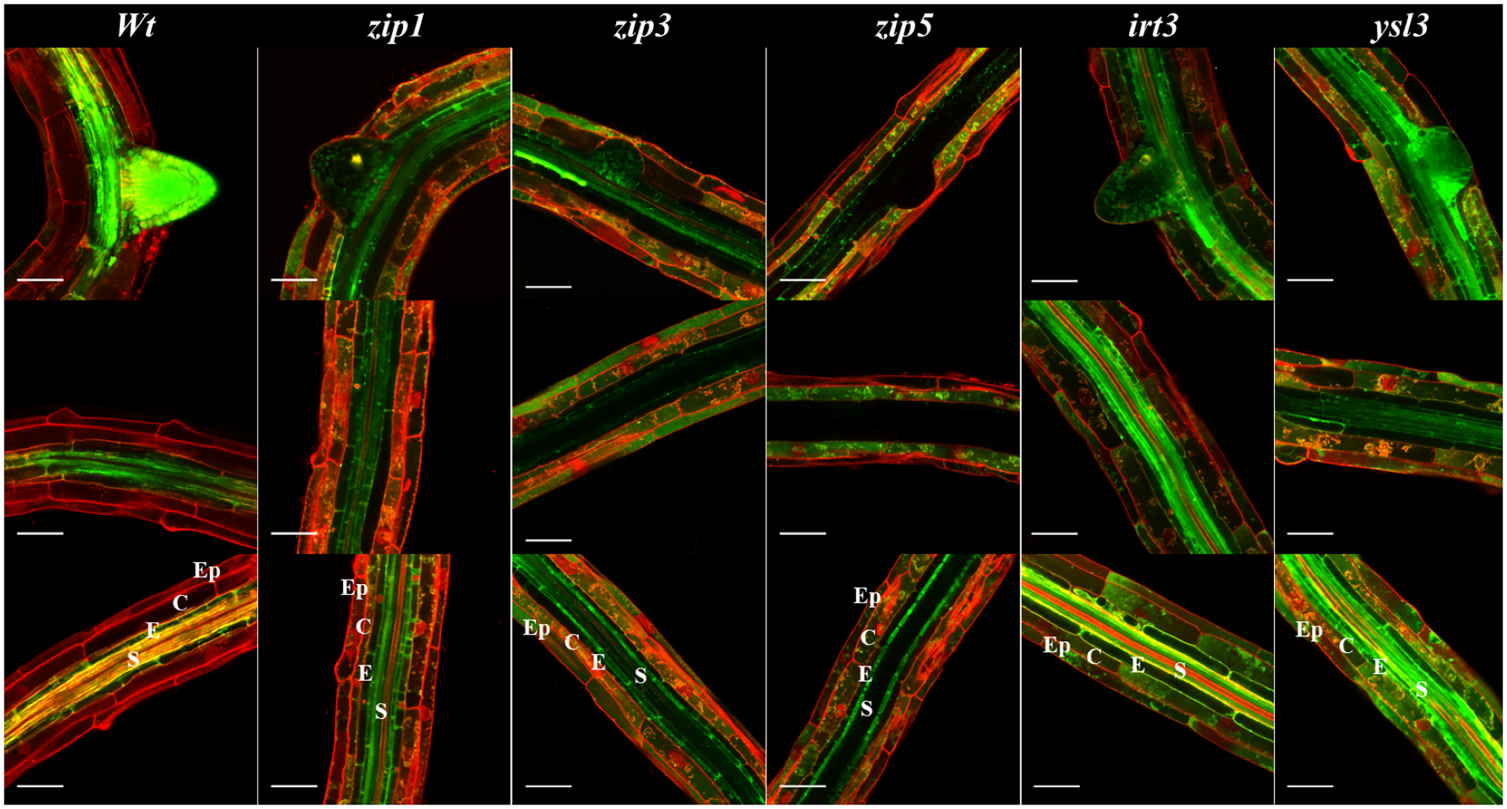
Confocal laser scanning microscope images of Zn distribution in *A. thaliana* wild type and Zn transporter gene mutants. Roots of Zn deficient *A. thaliana* seedlings are incubated with Zinpyr‐1 to detect Zn. Zinpyr‐1‐Zn complexes fluoresce green, propidium iodide mainly highlights cell walls as red fluorescence. Different regions from the older (top) to the younger (bottom) part of the root differentiation zone of five‐day‐old seedlings are shown for Col‐0 wild‐type, *zip1*, *zip3*, *zip5*, *irt3* and *ysl3* plants. The root cell layers are distinguished from the outside to inside as epidermis (Ep), cortex (C), endodermis (E) and stele (S) as indicated in the bottom panel of each column. The scale bars indicate 50 μm.

After establishing the Zn distribution in roots of wild‐type seedlings, we analyzed the Zn distribution in roots of several Zn transporter loss‐of‐function mutants. We chose to examine mutants for the *ZIP1, ZIP3, ZIP5, IRT3* and *YSL3* genes, which are all expressed upon Zn deficiency in roots (Table 1), and for which T‐DNA insertion mutants are available (Figure S2). The five‐day‐old *zip1, zip3, zip5, irt3* and *ysl3* mutant roots all show a Zinpyr‐1 fluorescence pattern similar to that of wild‐type plants (Figure 3; Figure S3), with some exceptions. In *zip1*, *zip3*, *zip5* and *irt3* roots, several lateral root primordia are noticeably low in Zinpyr‐1 fluorescence than wild type. The roots of *irt3* mutants show stronger Zinpyr‐1 fluorescence in stele, cortex and epidermis closer to the root tip. More fluorescence signal is also seen in the outer layers of the root, epidermis and cortex in the *ysl3* mutant (Figure 3). The *zip3* and *zip5* mutants show the strongest difference in Zinpyr‐1 fluorescence distribution compared to the wild type. Both mutants show very low Zinpyr‐1 fluorescence in the stele, similar to the seven‐day‐old wild‐type seedlings that experienced the strong Zn deficiency for 2 days longer (Figure 2). This suggests early Zn deficiency response to occur in these mutants. The Zn signal is predominantly seen in pericycle cells within the stele of the *zip3* and *zip5* mutants, but not in the whole stele as in the wild type. In the differentiation zone close to the root–shoot transition region, the Zn signal in cortex and epidermis in *zip3* mutants, and mainly in the cortex (and not the stele) in z*ip5* mutants, is in striking contrast with the mainly stele‐localized Zn signal of the wild type.

### Zn transporters are expressed in different cell layers of the root

The ZIP family of transporters is generally known to transport divalent cations (Fe^2+^, Zn^2+^, Cd^2+^, Ni^2+^ or alike) into the symplast, while the HMA and CDF family transporters are mostly thought to be involved in the export of cations from the symplast, either to the intercellular space or to the vacuole or other organelles (Eide, 2006; Eren & Argüello, 2004; Kobae et al., 2004; Sinclair & Krämer, 2012). We decided to study the root cell‐specific expression of several *ZIP*, *HMA* and *CDF* genes suggested to be implicated in Zn transport. For that purpose, we generated transgenic *A. thaliana* lines expressing a *super YELLOW FLUORESCENT PROTEIN 2* gene, cloned in frame with a nuclear localisation signal sequence (NLS‐*sYFP2*), of which transcription is controlled by the promoters of *ZIP1, ZIP3, ZIP5, ZIP9, ZIP10, ZIP11, ZIP12, IRT3, MTP2, YSL3* and *HMA2*. As a rule of thumb, we considered promoter regions of at most 1801 bp upstream of the predicted coding sequence start codon, but depending on the position of the upstream gene, this could be shorter. According to this consideration, *ZIP11* has the shortest promoter region, of only 426 bp. Three *ZIP* genes share their promoter regions with other genes: *ZIP1* with an auxin responsive gene (At3g12760) (Biswas et al., 2007); *ZIP9* with a sulfolipid synthetase (At4g33030) (Essigmann et al., 1998; Sanda et al., 2001); and *ZIP10* with a gene of unknown function (At1g31270).

**Figure 4:**
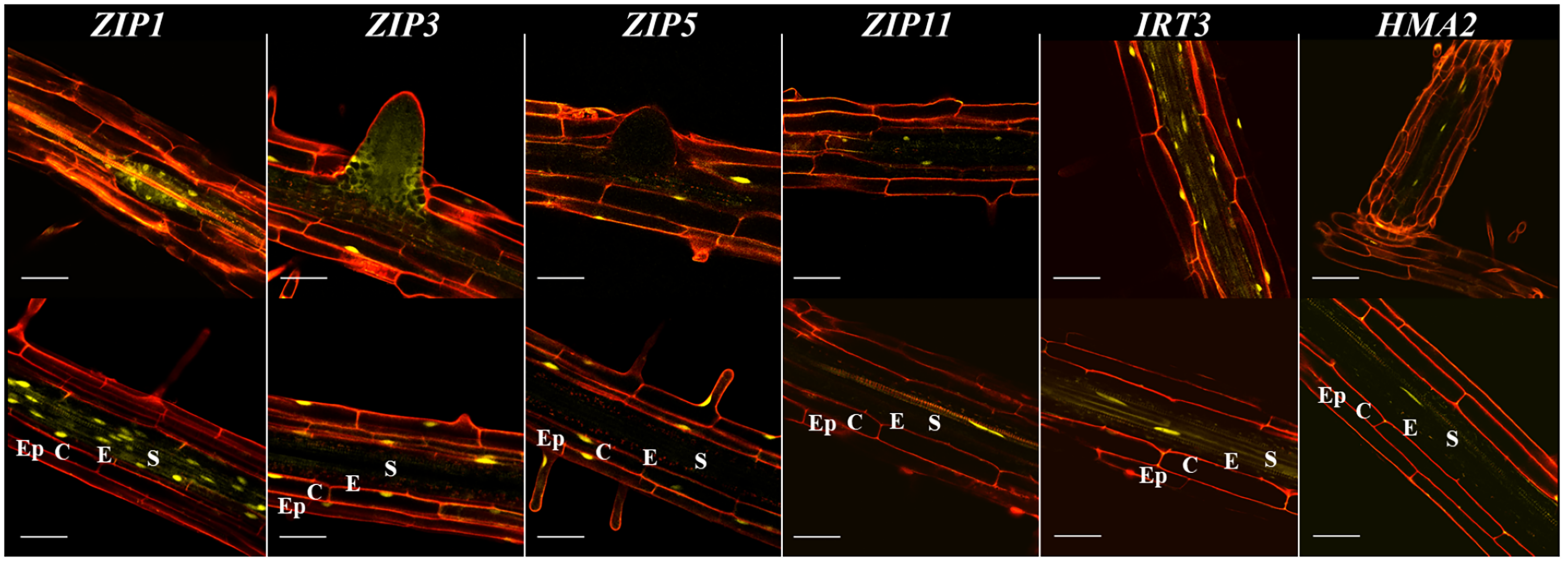
Confocal laser scanning microscope images of transgenic *A. thaliana* roots expressing nuclear‐localized super YELLOW FLUORESCENT PROTEIN 2 (sYFP2) driven by the promoter regions of indicated Zn transporter genes. The YFP fluorescent signal (yellow) can be seen in nuclei of cells in which a promoter is active. Propidium iodide is used to indicate cell walls (red fluorescence). Two zones are distinguished, and detailed for each Zn transporter promoter‐sYFP construct, from the older (top) to the younger (bottom) part of the root differentiation zone. The root cell layers are distinguished from the outside to inside as epidermis (Ep), cortex (C), endodermis (E) and stele (S) as indicated in the bottom panel of each column. Although the stele itself consists of different layers, the longitudinal images do not provide sufficient resolution to distinguish these. The scale bars indicate 500 μm in the whole root and 50 μm in the close‐up images.

For cell‐specific expression, we examined two independent transgenic lines per construct, which showed the same expression pattern. The expression was analyzed only in the primary root, except for *HMA2*. All promoters, except for *ZIP10*, induced YFP expression detectable by confocal microscopy (Figure 4; Figures S4 and S5). The *ZIP1* promoter is active in the endodermis, stele and lateral root primordia. *ZIP3* and *ZIP5* promoters are active in the epidermis and cortex, from the elongation zone to the root–shoot transition region. *ZIP3* is also highly active in lateral root primordia, where *ZIP5* is not active. *ZIP11* is active from the elongation to the root–shoot transition region. The location of *ZIP11* promoter activity changes along the root. It is active in xylem parenchyma in the young differentiation zone of the roots but in endodermal cells in the older part of the differentiation zone. The *IRT3* promoter is active from the root tip to the root‐shoot transition region. In the young differentiated zone, *IRT3* is active in the stele, while in the older differentiated zone, *IRT3* is active in all cell types from the epidermis to stele (Figure 4; Figure S4). The activity of the *ZIP9* and *ZIP12* promoters is only detected in the transition zone (Figure S5). There is little difference in YFP expression between the *ZIP9*‐ and *ZIP12*‐YFP transgenes, which suggests the transition zone YFP signal might not properly reflect promoter activity, as the expression of *ZIP9* is much higher than that of *ZIP12* (Figure 1). In a previous report, *pZIP9::GUS* shows expression in all cell types (Lee et al., 2021). We therefore did not include these transgenes in further analysis.

Zn efflux into the stelar apoplast, and eventually into the xylem, is mainly attributed to HMA2 and HMA4 (Hussain et al., 2004; Sinclair et al., 2007), with only *HMA2* expression found to be induced by Zn deficiency (van de Mortel et al., 2006). The *HMA2* promoter is active in xylem parenchyma (Figure 4) mainly in the young differentiated zone and in secondary roots. The *YSL3* promoter is active from the root cap to the root–shoot transition region. In the differentiated zone, *YSL3* is active in the epidermis and cortex; however, YFP expression is very hard to detect (Figure 4; Figure S4). The *MTP2* promoter is only active in the root–shoot transition region, particularly in the root hairs (Figure S5).

### Zn transporters are largely redundant in response to Zn deficiency

**Figure 5:**
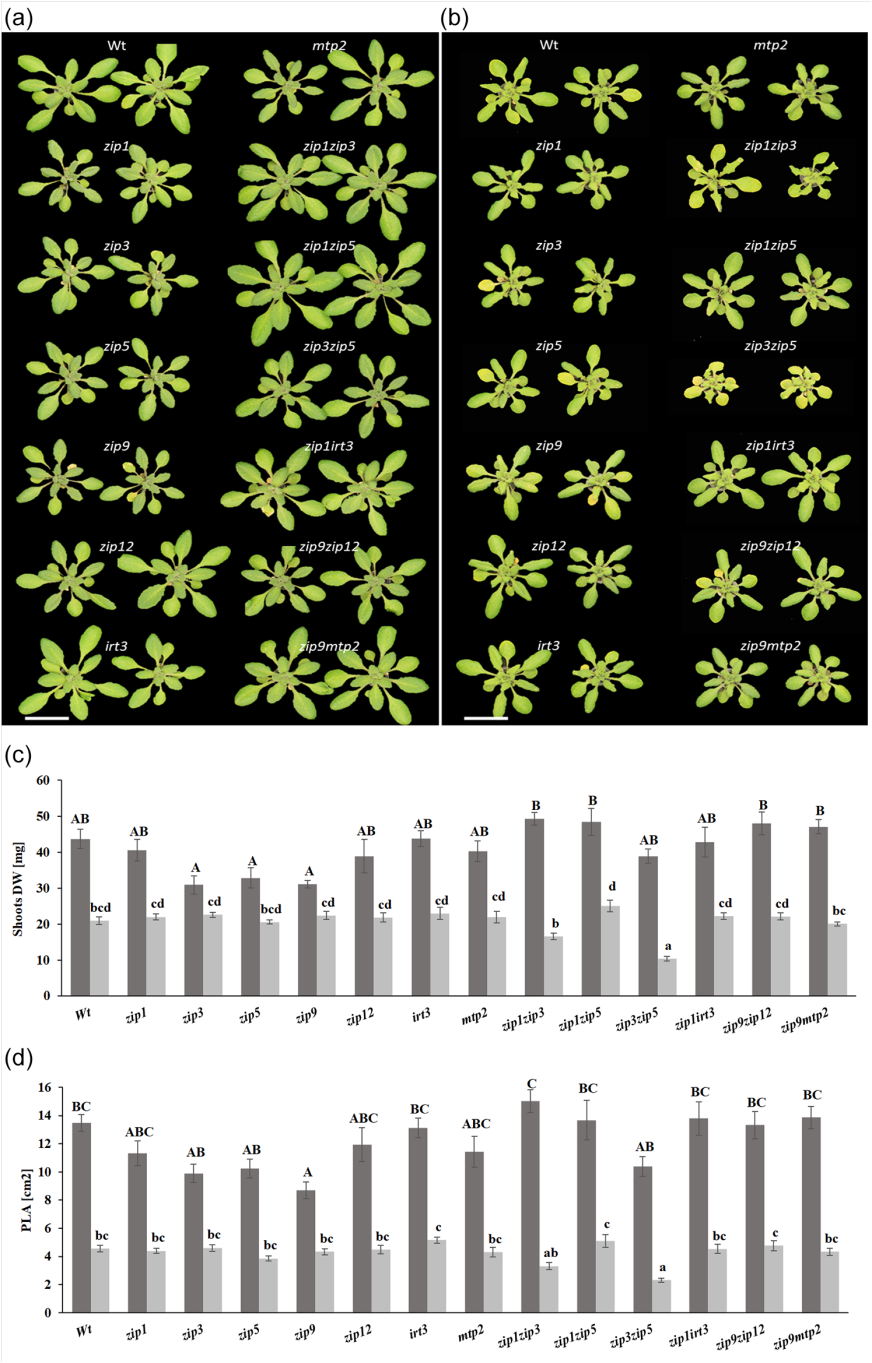
Response of single and double Zn transporter mutants grown under Zn sufficiency and Zn deficiency. Two representative plants of wild‐type, single and double mutant lines are shown that are grown hydroponically for (a) 25 days under Zn sufficiency or (b) 10 days under Zn sufficiency and 15 days under Zn deficiency. The scale bars indicate 2 cm in (a)–(b). (c) Shoot DW of wild‐type and Zn transporter mutant plants grown hydroponically for 25 days under Zn sufficiency (dark bars) or for 10 days under Zn sufficiency and 15 days under Zn deficiency (light bars). (d) The same as (c), but for projected leaf area. Mean ± SE, *n* = 10 plants. Upper case letters (Zn sufficiency) and lower case letter (Zn deficiency) above the bars denote statistically different groups, when comparing among genotypes grown under each treatment, obtained with a Tukey post hoc test (*α* = 0.05), after a one‐way ANOVA (*P* < 0.01).

Plant response to the loss of one or two *ZIP* transporters is evaluated in transporter mutants grown in Zn sufficiency and Zn deficiency. In Zn sufficiency, the *zip1*, *zip3*, *zip5* and *zip9* mutants are noticeably smaller than the wild type (Figure 5a), and though they have a lower shoot dry weight (DW) and projected leaf area than wild‐type plants, this difference is only significant for the projected leaf area of the *zip9* mutant (Figure 5d). In Zn deficiency, the shoot phenotypes of most mutants cannot be distinguished from the wild type. Only the double mutant *zip3zip5* is noticeably smaller, with more yellow and curled leaves than the wild type (Figure 5b), with also a significantly lower shoot DW and projected leaf area than that of the wild type (Figure 5c,d). The root DW data show the same trends, but the observed differences are not significantly different from the wild type (Figure S6).

**Figure 6:**
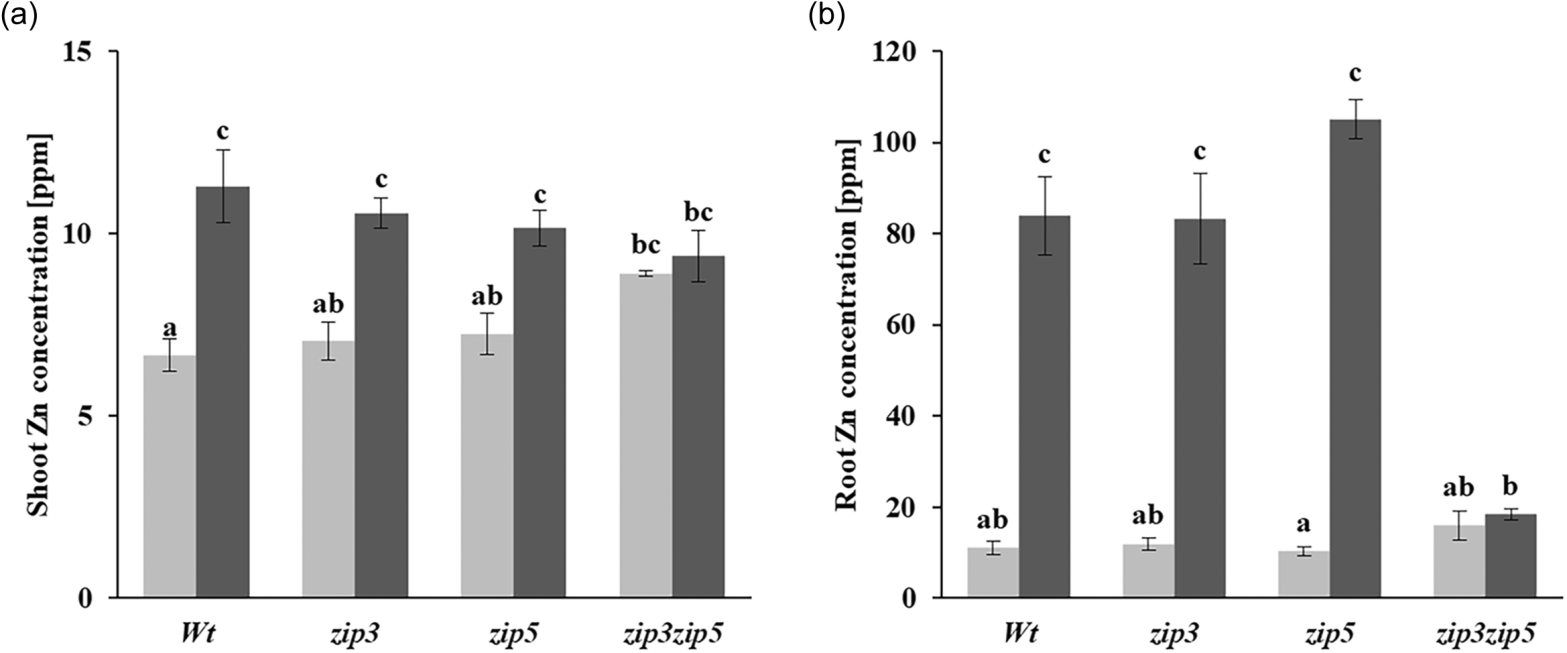
Zinc concentrations of Zn deficient *zip3* and *zip5* single, and *zip3zip5* double mutant before and after being resupplied with Zn. Plants are grown in soil for 15 days and thereafter for 30 days on Zn deficient hydroponic medium (light bars), or re‐supplied with Zn for 3 h (dark bars), and sampled for Zn concentration measurements. Mean ± SE are shown of *n* = 6 plants. Letters above the bars denote statistically significantly different groups, as determined with a Tukey post hoc test (*α* = 0.05), after a two‐way ANOVA (*P* < 0.01 for genotype × treatment interaction).

As the *zip3zip5* double mutant is the only one with a significantly lower shoot DW compared to the wild type in Zn deficiency, we compared its Zn uptake and translocation capacity upon resupply with Zn to that of the wild type and both single *zip3* and *zip5* mutants. Zn‐deficient plants are supplied with 2 μM ZnSO_4_ for 3 h before their Zn concentration is determined (Figure 6). As expected, the Zn concentration of all plants is very low in shoots and roots of Zn deficient plants, although the shoot concentration of the *zip3zip5* double mutant is significantly higher than that of the wild type. Three hours after resupply of Zn, the Zn concentration has increased significantly in shoots and roots of nearly all genotypes. The increase in Zn concentration is particularly strong in roots, with a more than 10‐fold increase in the wild type and both single *zip3* and *zip5* mutants. Only for the *zip3zip5* double mutant, the resupply of Zn does not significantly increase in Zn concentration, not even in the roots, which would be expected to show the fastest increase due to direct uptake. The inability to absorb noticeable amounts of the resupplied Zn in the *zip3zip5* double mutant means that ZIP3 and ZIP5 have an essential, but mutually redundant, function in the uptake of Zn by the root.

### Shoot and root ionome profiles of especially double mutants deviate from wild type

**Figure 7:**
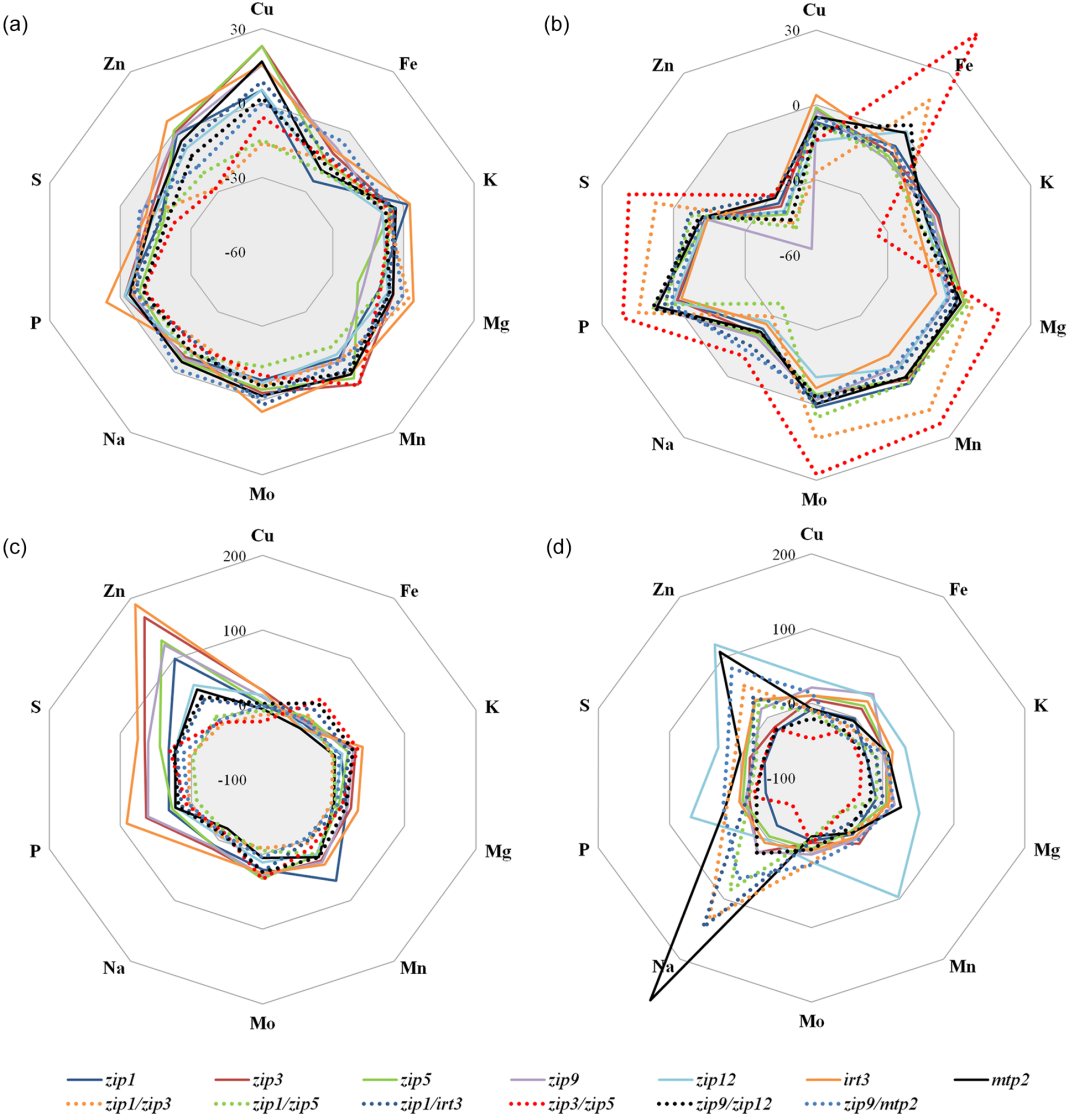
Overview of ionomic profiles of *A. thaliana* single and double Zn transporter mutants grown under Zn sufficiency or Zn deficiency. Graphs present the percentage of difference of Cu, Fe, K, Mg, Mn, Mo, Na, P, S, and Zn concentration in shoots (a, b) and roots (c, d) of the mutants compared with wild‐type plants, calculated as {([Mutant] – [Wt])/[Wt]} × 100. Plants are grown hydroponically for 25 days under Zn sufficiency (a, c) or for 10 days under Zn sufficiency and 15 days under Zn deficiency (b, d). Mineral concentrations are presented in Figure S7.

In an additional experiment, the shoot and root ionome profiles of the single and double Zn transporter mutants were determined and compared to those of wild‐type plants, grown under Zn sufficiency or Zn deficiency (Figure 7; Figure S7). Upon Zn sufficiency, shoots of all single and double mutants contain less Fe, and S, while less Zn is mainly observed in the double mutants, particularly in *zip1zip3* and *zip3zip5* (Figure 7a). This lower Zn concentration is not seen in the roots, on the contrary. In the roots, all but the *zip1zip3*, *zip1zip5*, *zip1irt3* and *zip3zip5* mutants show a much higher Zn concentration, with over twice as much Zn in the *irt3*, *zip3*, *zip5*, *zip9* and *zip1* mutants. Several single mutants also show higher P and S concentrations (Figure 7c). The shoot ionome profiles of mutants grown in Zn deficiency are strikingly different from wild type. In general, all mutants have lower concentrations of K, Na, and Zn, with the lowest Zn concentration found in the *zip9* mutant. The *zip1zip3* and *zip3zip5* mutant profiles are most deviating from wild type, with the lowest K and the highest Fe, Mg, Mn, Mo, P, and S concentrations (Figure 7b). The roots of several mutants have higher Na and Zn concentrations, especially *mtp2* (Figure 7d). Also, the *zip1* double mutants (*zip1zip3*, *zip1zip5*, and *zip1irt3*) and the *zip9mtp2* double mutant have (much) higher Na concentrations than wild type. The *zip12* mutant shows remarkably high concentrations of Zn, Mn, and P, but also several other element concentrations are higher than in wild type. The higher concentrations in roots and lower concentrations in shoots generally result in a strongly reduced root‐to‐shoot translocation of particularly Zn and Na in mutants compared to wild‐type plants, under both Zn supply conditions. Very different in this respect is the *zip3zip5* double mutant when exposed to Zn deficiency, which has a significantly higher translocation for Cu, Fe, Mg, Mn, Mo, Na, P and S than the wild‐type plants (Figure S8).

### Loss of Zn transporter gene function may be compensated by higher expression of other Zn transporter genes

**Figure 8:**
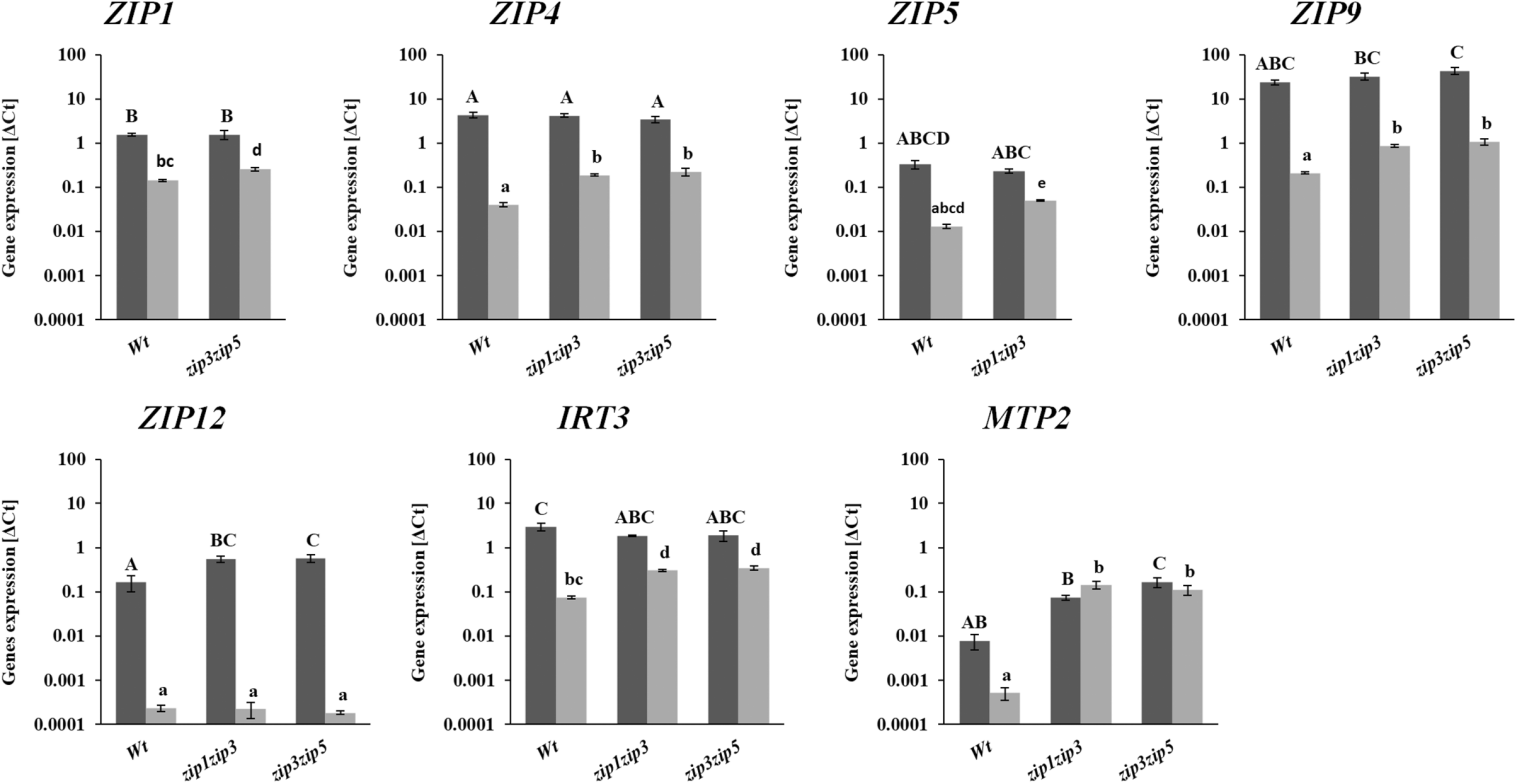
Normalized Zn transporter gene expressions in *A. thaliana* double Zn transporter mutant plants grown under Zn deficiency. Plants are grown hydroponically for 10 days under Zn sufficiency and 12 days under Zn deficiency. Gene expression of shoots (dark bars) and roots (light bars) is normalized to the expression of two reference genes (At5g25760 and AT2G28390). Average normalized expressions ± SE are shown on a ^10^log‐scale, *n* = 3 samples of 2 pooled plants each. Upper case letters (shoot) and lower case letters (root) above the bars denote statistically different groups, when comparing among genotypes grown under each treatment, obtained with a Tukey post hoc test (*α* = 0.05), after a one‐way ANOVA (*P* < 0.01). Full set of genes and genotypes tested are detailed in Figure S9.

The expression of nine Zn transporter genes is determined in each single and double mutant, to establish if loss of Zn transporter function is compensated by enhanced expression of other transporter genes upon Zn deficiency. For the single mutants, transporter gene expression, other than that of the mutant gene, is comparable to that of the Col‐0 wild type (Figure S9). Double mutants, however, indeed show higher expression of some Zn transporter genes upon Zn deficiency. In *zip1zip3* and *zip3zip5* double mutants, *ZIP4*, *ZIP9*, *IRT3* and *MTP2* are higher expressed in roots and *ZIP12* in shoots. In addition, *ZIP5* is higher expressed in *zip1zip3* roots, *ZIP1* in *zip3zip5* roots and *MTP2* in *zip3zip5* shoots (Figure 8).

## DISCUSSION

### The gene expression response to Zn deficiency starts in roots

When plants face nutrient deficiency, a strategy for survival is to allocate available nutrients to metabolically active parts. In aerial parts, nutrients may be remobilized from older leaves and stem via the phloem to nutrient requiring parts, such as meristems and young leaves or flowers (Diaz‐Mendoza et al., 2016; Maillard et al., 2015). Alternatively, nutrients may be taken up from the soil by the roots and subsequently transported through the vasculature to the metabolically active parts. The latter strategy appears to be preferred by *A. thaliana*, at least for allocating nutrients to seeds (Waters & Grusak, 2008). Both strategies rely on changes in gene expression (Maillard et al., 2015). The plant transcriptome is very flexible and constitutes a key factor to respond rapidly to environmental changes, such as imminent nutrient deficiency (de Nadal et al., 2011).

**Figure 9:**
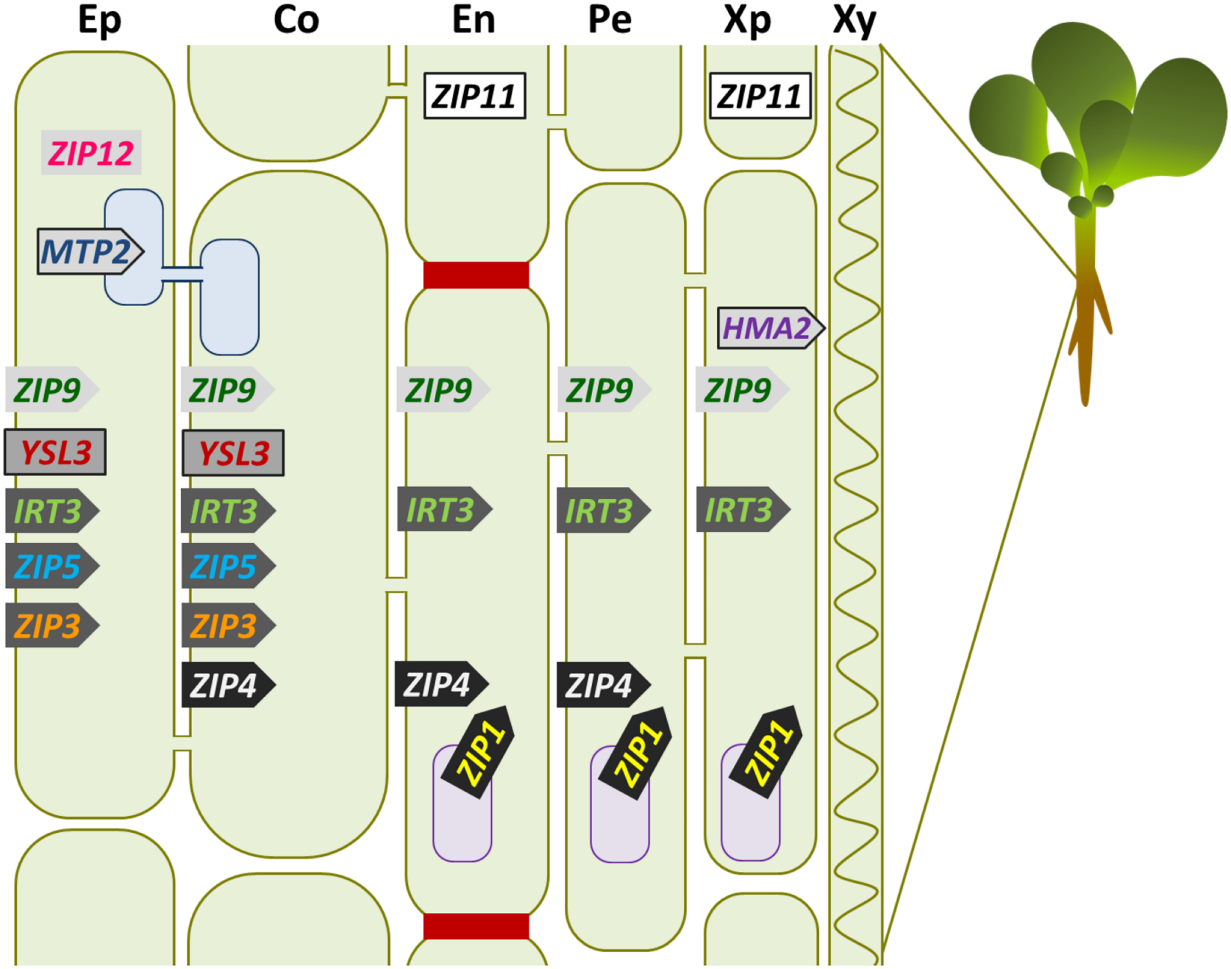
Schematic overview of Zn transporters in roots upon Zn deficiency. Root cell layers can be distinguished from the outside to inside as epidermis (Ep), cortex (Co), endodermis (En), pericycle (Pe), xylem parenchyma (Xp), and xylem (Xy). The earlier the gene is expressed in response to Zn deficiency, the darker the fill of the arrow or box symbol indicating the Zn transporter. Zn transporters symbols without border indicate those that are under transcriptional control of bZIP19/bZIP23. Arrows indicate the direction in which Zn is transported. Vacuoles are indicated as purple ellipses, the endoplasmic reticulum is indicated as blue connected ellipses. Casparian strips between endodermis cells block apoplastic transport into the stele.

Based on the promoter‐YFP expression data obtained in this study and on previous Zn transporter studies, we propose a model for the action of Zn transporters in roots, largely building on the model proposed by Sinclair and Krämer (2012) (Figure 9). The Zn deficiency response gene expression cascade is initiated and mediated by the transcription factors bZIP19 and bZIP23 (Assunção et al., 2010). Recently was shown that these two bZIP transcription factors also act as nuclear sensors of Zn deficiency by binding Zn (Lilay et al., 2020). *A. thaliana* plants thus are well able to sense Zn deficiency and act by expressing Zn transporter genes. It is not clear yet if the Zn sensing signal is first perceived in shoots, and then transferred to roots, or that the reduced uptake of Zn is first detected in roots, where it activates the Zn deficiency response. However, we show that the first signs of Zn transporter gene expression occur in roots, within 6 h after exposure to Zn deficiency (Table 1). The first genes to show a significant induction of transcription are *ZIP1* and *ZIP4*, later followed by *ZIP3*, *ZIP5* and *IRT3*, suggesting that these are most important in restoring plant Zn status. They are all likely to transport metal ions into the symplast (Eide et al., 1996).

*ZIP1* and *ZIP4* are the first to be induced, which, based on what we know so far of both genes, will overcome immediate bottlenecks in Zn import into the stele. Plasma membrane‐localized ZIP4 covers import of apoplastic Zn into cortex, endodermis and pericycle cells (Lin et al., 2016), which is likely to ensure the initial additional Zn uptake into the stelar symplast. Tonoplast‐localized ZIP1 will aid in releasing stored Zn from the tonoplasts (Milner et al., 2013) of endodermis, pericycle and xylem parenchyma cells to move it into the stele. Subsequently, the expression of *ZIP3*, *ZIP5* and *IRT3* is induced. Together with *ZIP1* and *ZIP9*, *ZIP3* and *IRT3* are also the highest expressed Zn transporter genes under Zn sufficient conditions (Assunção et al., 2010; Inaba et al., 2015; van de Mortel et al., 2006) (Figure 1), and likely constitute the regular Zn uptake machinery. ZIP3 and ZIP5 are expressed in the epidermis and cortex, which in *A. thaliana* hold most of the high‐affinity nutrient uptake transporters, such as the high‐affinity SULTR1.2 sulphate transporters (Takahashi, 2019), the high‐affinity NRT1/NRT2 nitrate transporters (Wang et al., 2012), the major PHT1 phosphate uptake transporters (Mudge et al., 2002) and the high‐affinity IRT1 iron uptake transporter (Vert et al., 2002). We therefore also expect ZIP3 and ZIP5 to be high‐affinity Zn uptake transporters.

The epidermis and cortex are functionally similar in terms of nutrient absorption (Barberon & Geldner, 2014). The endodermis basically blocks apoplastic transport due to the Casparian strips and subsequent suberization blocks nutrient uptake through plasma membrane ion transporters (Barberon et al., 2016), leaving the symplastic pathway for nutrients to access from the cortex through the endodermis into the stele (Barberon & Geldner, 2014; Geldner, 2013). *IRT3* is co‐expressed with *ZIP3*, but also in the endodermis and the stele. IRT3 is plasma‐membrane localized (Lin et al., 2009), and together with ZIP4, provides additional opportunity to mop up any available apoplastic Zn, not taken up by ZIP3 or ZIP5, and keep it in the symplast for further transport into the stele. We did not investigate the subcellular location of these proteins, however *OsZIP5* and *OsZIP9*, orthologues of *AtZIP5* and *AtZI9* respectively, are localized at the plasma membrane (Tan et al., 2020).

After the initial induction of the Zn deficiency response, the expression of ZIP1, ZIP3, ZIP4, ZIP5 and IRT3 remains high in Zn deficient roots (Table 1; Figure S1), and for nearly all tested genes is highest in roots and shoots at Day 12 (Figure 1). The only exceptions are *HMA2* in shoots, *ZIP11* in roots, and *YSL3*. With *MTP2*, these genes lack the ZDRE motif in their promoter, which is present in the promoters of the other *ZIP‐*family genes, and which is the target sequence for binding the bZIP19 and bZIP23 transcription factors (Assunção et al., 2010). Expression of *HMA2*, *MTP2*, *ZIP11* and *YSL3* are likely to constitute a secondary or tertiary response to Zn deficiency, not controlled by bZIP19/bZIP23. HMA2 and MTP2 are involved in partitioning Zn from roots to shoots (Hussain et al., 2004; Sinclair et al., 2007; Sinclair et al., 2018) while the plasma membrane‐localized YSL3 (Chu et al., 2010) is more likely to transport nicotianamine‐chelated Zn (Curie et al., 2009; Waters et al., 2006). Previously, MTP2 expression was found in the endoplasmic reticulum of secondary roots (Sinclair et al., 2018), while in our analysis, the activity of the *MTP2* promoter was only detected in the root hairs in the transition zone (Figure S5), probably because secondary roots were only just emerging.

### Loss‐of‐function of Zn transporters affects Zn distribution in A. thaliana roots

Knowing the site of Zn transporter expression, we determined how this corresponds with the presence of Zn in Zn‐depleted seedling roots by tracing Zn distribution. The Zinpyr‐1 fluorescence in these roots was intense in zones with many dividing cells, such as the root tip and lateral root primordia, suggesting that during Zn shortage, Zn is allocated to metabolically active cells (Avice & Etienne, 2014; Himelblau & Amasino, 2001). The region just above the root meristem is also an easy, and uncontrolled, access point of minerals into the stele as neither Casparian strips nor suberin layers are deposited yet (Barberon et al., 2016; Geldner, 2013). In the root differentiation zone, Zn is mostly restricted to the stele, and gradually disappears upon longer Zn deficiency exposure, to become further restricted to the younger part of the stele. When moving up the root, Zn is prominently detected again in the root–shoot transition region, between primary root and hypocotyl, where the vasculature organization is rearranged, and nutrient metal leakages may occur. Zn storage in the root is thus rapidly reduced upon Zn deficiency, favoring mobilization into the stele for further transport to the regions that are in most demand of Zn, very much in line with the observed gene expression patterns of Zn transporter genes.

Even though there appears to be high redundancy among Zn transporters, we expected to see the effect of Zn deficiency on root Zn distribution in transporter mutants. Such was the case, but the effect was subtle. The root Zn distribution pattern of five‐day‐old *zip1, zip3*, *zip5* and *ysl3* mutants resembles that of seven‐days‐old wild‐type seedlings, which means these mutants become Zn deficient only a few days sooner than the wild type (Figure 3; Figure S3). The strongest effect is seen in the *zip3* and *zip5* mutants. In both mutants, there is hardly any Zn detected in the stele of the older parts of the differentiation zone, though it is still present in cortex, epidermis, and pericycle. The radial transport of Zn into the stele is obviously diminished in these mutants, leading to even faster depletion of Zn in the shoot upon Zn deficiency. The Zn stele depletion seems to be a common pattern among Zn transporter mutants as seen before for triple and quadruple mutans (Lee et al., 2021).

While the short‐term effect of Zn deficiency on young mutant seedling roots is subtle, the long‐term effect of transporter mutations on the mature plant ionome is considerable (Figure 7). At Zn sufficiency, the *zip1, zip3, zip5, zip9* and *irt3* mutants accumulate Zn in their roots, illustrating their difficulty in maintaining Zn homeostasis. At Zn deficiency, most mutants retain Zn in the roots, more or less at wild‐type levels, but show strong Zn depletion in the shoots, most prominently in the *zip9* mutant. Especially, the *zip12* and *mtp2* mutants accumulate more Zn in roots than the wild type. For the *mtp2* mutant, this is not surprising, as it has already been implicated to function in root‐to‐shoot Zn translocation. MTP2 is proposed to contribute to the radial symplastic transport of Zn to the stele, particularly by accommodating plasmodesmatal Zn transport (Sinclair et al., 2018). The Zn accumulation in *zip12* roots is surprising, as *ZIP12* is not highly expressed in *A. thaliana*, even though expression is highly induced by Zn deficiency (Figure 1). It could be this gene is expressed in only few root cells. A candidate location for such expression would be in the passage cells. These constitute rare openings in the suberized endodermis of the older roots, that allow nutrient uptake in the differentiation zone and above (Peterson & Enstone, 1996).

### Zn transporters act redundantly

Zn deficiency reduces the biomass production of *A. thaliana* plants (Campos et al., 2017). We show that such reduction in biomass is even higher when the Zn uptake machinery is severely disturbed, but only in case at least two genes are mutated. In previous reports, single mutants of *ZIP5* (Wu et al., 2009), *ZIP12* (Inaba et al., 2015), and *OsZIP5* (Lee et al., 2010; Tan et al., 2020), and single and double mutants of *ZIP4*, *ZIP6*, *ZIP9* and *IRT3* (Lee et al., 2021) exhibited a slight or not detectable mutant phenotype. Whereas the double mutants *oszip5oszip9* (Tan et al., 2020) or the triple mutants *irt3zip4zip6* and *irt3zip4zip9* showed detectable mutant phenotypes (Lee et al., 2021). This means there is a high level of functional redundancy among Zn transporters, of which we only reveal redundancy of *ZIP1*, *ZIP3* and *ZIP5*. When compared to wild type, the further reduction in biomass in the *zip3zip5* and *zip1zip3* double mutants is significant and substantial (Figure 5). *zip3zip5* is the most sensitive mutant to Zn deficiency, with smaller rosettes, curly, and more yellow leaves and much lower shoot biomass than wild‐type plants. It easily stands out as the most deviant rosette phenotype of all single and double Zn transporter mutants we tested. *ZIP3* and *ZIP5* are phylogenetically closely related (Mäser et al., 2001), both are expressed in the root epidermis and cortex, and both share a similar mutant Zn distribution pattern, so likely they perform the same function. By abolishing the function of both genes, the *zip3zip5* mutant is severely impaired in its main Zn acquisition route. This also explains why the expression of *ZIP4* and *IRT3*, and probably also *ZIP9*, is increased in the *zip3zip5* double mutants compared to wild type (Figure 8). In the absence of *ZIP3* and *ZIP5*, expression of these Zn transporters is essential to move apoplastic Zn into the symplast. The similarly increased expression of these genes in the *zip1zip3* double mutants (Figure 8) suggests the Zn remobilization from root vacuoles by ZIP1 to be of comparable importance to support apoplastic Zn acquisition through ZIP5. These two double mutants are also the only two that accumulated several other elements, particularly Fe, Mg, Mn, Mo, P and S, to higher levels in the shoot than found for wild type. We did not examine this any further, but this is likely to be a consequence of the shortage of Zn in the stele of the root, which may enhance the expression of transporters more prone to transport other elements, but with low affinity to also transport Zn, such as IRT1 (Korshunova et al., 1999), IRT2 (Vert et al., 2001), NRAMP1 (Cailliatte et al., 2010) or YSL1 (Waters et al., 2006). While we expected to reveal more *ZIP* gene redundancies, that was not the case. Likely suspects to try are all combinations of the early and medium responding genes, *ZIP1, ZIP3, ZIP4, ZIP5, ZIP9* and *IRT3*. As all of these genes are under control of the bZIP19/bZIP23 transcription factors, increasing loss of Zn transporter activity is expected to ultimately resemble the extremely Zn deficiency sensitive phenotype of the *bzip19bzip23* double mutant (Assunção et al., 2010).

## EXPERIMENTAL PROCEDURES

### Plant material

T‐DNA insertion lines for the studied *A. thaliana* genes were ordered from the Nottingham Arabidopsis Stock Centre (www.arabidopsis.info) (Figure S2 and Table S1). *A. thaliana* accession Columbia (Col‐0) was used for transformation and as a wild‐type control.

### Growing conditions

Prior to germination, seeds were stratified for 3 days at 4°C in the dark, upon which germination was thought to start. For propagation, genotyping, and the Zn uptake experiment plants were grown in a fertilized peat mixture in a glasshouse set at a 16 h light/8 h dark photoperiod, 20°C/18°C (day/night) temperature, and 70% humidity. For the Zn uptake experiment, plants grew for 15 days in a fertilized peat mixture. Plants were subsequently uprooted and grown hydroponically for 30 days in Zn deficiency (no Zn added to the medium). Thereafter, Zn‐starved plants were re‐supplemented with Zn for 3 h by transferring them to medium containing 2 μM ZnSO_4_.

For tissue culture, seeds were surface‐sterilized using vapour‐phase seed sterilization (Clough & Bent, 1998). For Zn deficiency experiments, seeds were sown on 0.55%‐agar‐filled tubes in trays containing a modified half‐strength Hoagland's nutrient solution (Schat et al., 1996). Plants were grown hydroponically in a controlled growth cabinet set at 12/12 h light/dark, 20°C/15°C (day/night) and 70% humidity. For phenotyping Zn deficiency, plants grew for 10 days in a fully supplemented medium (containing 2 μM ZnSO_4_), after that, plants were either treated with Zn deficiency (no Zn added to the medium) or continued to grow at fully supplemented medium. Plants used for gene expression grew for 15 days (time points experiment) or 10 days (Zn transporter mutants experiment) in a fully supplemented medium (containing 2 μM ZnSO_4_), after that, plants were either treated with Zn deficiency (no Zn added to the medium) or continued to grow in fully supplemented medium.

For the Zn tracking and gene expression imaging, plants were grown in a controlled growth cabinet set at 16/8 h light/dark, 22°C/20°C (day/night), and 50% humidity. For Zn tracking, seeds were sown on 9‐cm Petri dishes filled with distilled‐water. For gene expression imaging, seeds were sown on 12‐cm‐square plates containing 1%‐agar‐solidified half‐strength MS Zn deficiency medium (no Zn added to the medium) (pH 5.8). Plates were placed vertically for plant growth.

### Genotyping

Homozygous T‐DNA insertion lines were selected by PCR. For *ZIP1*, *ZIP3*, *ZIP5*, *ZIP9*, *MTP2* and *YSL3*, homozygous lines were found, with T‐DNA insertions in the transcribed region, for *ZIP12* and *IRT3*, T‐DNA insertions are located upstream of the predicted ATG start codon. Several of these T‐DNA insertion lines have already been confirmed to be mutants: *zip1* (Milner et al., 2013), *zip5* (Wu et al., 2009), *zip12* (Inaba et al., 2015), *zip9*, *irt3* (Lee et al., 2021), *mtp2* (Sinclair et al., 2018) and *ysl3* (Chen et al., 2011). Oligonucleotide primers were designed using the Salk Institute Website (signal.salk.edu/tdnaprimers.2.html). Two PCRs were performed per plant, one with the left border (LB) and T‐DNA border (BP) primers, the other with the LB and right border (RB) primers (Table S1). Double knock‐out mutants were generated by crossing single knock‐out mutants and selection by PCR of the double knock‐out mutants among the F2 progeny, which was confirmed in F3.

### Zn tracking

Five‐ to seven‐day‐old seedlings were incubated with 5 μM of Zinpyr‐1 (Sigma‐Aldrich; www.sigmaaldrich.com) for 3 h, as described by Sinclair et al. (2007). Cell walls were stained with 10 μg mL^−1^ propidium iodide (PI) for 30 s. Images were taken with a Leica SPE DM5500 Confocal Laser‐Scanning Microscope (CLSM) based on an upright run with the LAS AF 1.8.2 software (Leica; www.leica‐microsystems.com). Zinpyr‐1 and PI were excited with a solid‐state laser at 488 nm. The Zinpyr‐1 and PI emissions were detected at a bandwidth of 540–550 and 700–800 nm respectively. Images were processed using Image J (Schneider et al., 2012).

### Gene expression analysis

For the time‐point experiment, the Zn deficiency treatment was applied on 15‐day‐old seedlings, starting 1 h after lights‐on. Samples were collected 15 min, 1, 3, 6, 12, 36 h, 4.5 and 12.5 days thereafter. Full root systems and shoots of three plants were collected for each sample. Three samples were collected at each time point for each treatment. Total RNA was extracted with a Direct‐Zol™ RNA MiniPrep Kit (Zymo Research, www.zymoresearch) and cDNA was synthetized with the iScript cDNA Synthesis Kit (Bio‐Rad, www.bio‐rad.). Gene expression was quantified by reverse transcriptase quantitative PCR (RT‐qPCR) using the SYBR^®^ Green mix (Bio‐Rad, www.bio‐rad.com). Genes and primers are listed in Table S2. Ct values of each gene were normalized to the reference genes *PEX4* (At5g25760) and *SAND* (At2g28390) (Campos et al., 2017). The relative expression values were calculated based on the ΔCt and ΔΔCt methods (Livak & Schmittgen, 2001). For single and double mutant plants, Zn deficiency treatment was applied on 10‐day‐old seedlings for 12 days. Full root systems and shoots of two plants were collected for each sample. Three samples were collected of each genotype. RNA extraction, gene expression quantification and normalization to reference genes was performed as mentioned above.

### Generation of promoter‐NLS‐YFP transgenic lines

Promoter sequences were amplified from *A. thaliana* Col‐0 by PCR. Promoter PCR fragments were first cloned into the pDONOR201 vector and subsequently into the destination vector, using Invitrogen Gateway technology. To create the destination vector, pFAST‐R07 (Shimada et al., 2010) was digested with *Eco*RI and *Nru*I to remove the eGFP sequence. In parallel, the *super YELLOW FLUORESCENT PROTEIN 2* (*sYFP2*) gene (Kremers et al., 2006) was PCR‐amplified from pCZN633 (Smaczniak et al., 2012) using primers containing a short linker including a *Nru*I restriction enzyme site and an overlapping sequence with a nuclear localisation signal (NLS) fragment. The NLS sequence was PCR‐amplified from pGREEN:GW:NLS‐GFP (Horstman et al., 2015) using primers containing a short linker including an overlapping sequence with the sYFP2 DNA fragment. The NLS and sYFP2 fragments were connected with a double joint PCR (Yu et al., 2004), subsequently digested with *Eco*RI and *Nru*I and ligated into the digested pFAST‐R07 resulting in the pFAST‐R7‐VO destination vector (Figure S10). Promoter sequences were cloned in pFAST‐R7‐VO by standard Invitrogen Gateway BP and LR reactions. Vector construction primers are detailed in Table S3. We aimed to amplify a promoter region of about 1800 bp upstream of the start codon of the target gene, but excluding the start or stop codon of the gene upstream of the target gene. Promoter cloning primers and sizes of promoter fragments are detailed in Table S4. Promoters were selected to be 426–1801 bp. *A. thaliana* Col‐0 plants were *Agrobacterium tumefaciens*‐transformed using the floral dip method (Clough & Bent, 1998). The transgenic T1 seeds were selected based on the ‘red seed’ fluorescence marker (Shimada et al., 2010).

### YFP fluorescence imaging

Homozygous or heterozygous transgenic T2 seeds were selected based on ‘red seed’ fluorescence (Shimada et al., 2010). Seedlings of two independent transgenic lines per construct were grown on Zn‐deficient vertical agar plates. Roots from six‐ to 10‐day‐old seedlings were PI‐stained and CSLM images were taken as described under *Zn tracking*. A solid‐state laser at 488 nm was used to excite sYFP2 and PI. The sYFP2 and PI emission were detected at a bandwidth of 530–570 and 700–800 nm respectively. Images were processed using Image J (Schneider et al., 2012).

### Metal concentration analysis

Shoot and roots were washed with MQ water, 1 mM EDTA (pH 8) and MQ water. For ionome profiling, plant material was collected in paper bags and dried for 3 days at 60°C. The elemental content was determined by ICP‐MS (NexION 2000), Perkin Elmer following the method adapted from Danku *et al*. (2013). For the Zn uptake assay, samples were dried overnight at 70°C, digested in Teflon bombs at 140°C for 7 h with a mixture of HNO_3_ (65%) and HCl (37%). The Zn concentration was determined using flame atomic absorption spectrometry (Perkin Elmer 1100B).

### Statistical analysis

Data were Log10‐transformed prior to any test to approach normality. Two‐sample t‐test, one‐ and two‐way ANOVA and Tukey post hoc tests were performed using Genstat 18th edition (VSN International; www.vsni.co.uk/software/genstat/).

## Author Contributions

The experimental design, the execution of the experiments and the writing of a draft manuscript was performed by Valeria Ochoa Tufiño under the supervision of Mark G.M. Aarts. Maria Almira Casellas and Aron van Duynhoven, contributed to the generation of the transgenic reporter lines. The Zn uptake experiment was performed by Henk Schat. Paulina Flis and David E. Salt performed the ionome analyses of the samples provided. All authors contributed to writing the final version of the manuscript.

## Conflict of Interest

The authors have not declared a conflict of interest.

## Supporting information


**Figure S1.** Normalized gene expression in shoots and roots of *Arabidopsis thaliana* in response to Zn deficiency at increasing time points.
**Figure S2.** Schematic drawings of Zn transporter genes with T‐DNA insertion.
**Figure S3.** Confocal laser scanning microscope images of Zn distribution in *Arabidopsis thaliana* roots of wild‐type and Zn transporter mutant lines.
**Figure S4.** Confocal laser scanning microscope images of *Arabidopsis thaliana* seedlings expressing nuclear‐localized YELLOW FLUORESCENT PROTEIN 2 (sYFP2) driven by the promoter regions of Zn transporter genes.
**Figure S5.** Confocal laser scanning microscope images of *Arabidopsis thaliana* seedlings expressing nuclear‐localized YELLOW FLUORESCENT PROTEIN 2 (sYFP2) driven by the promoter regions of Zn transporters *ZIP9*, *ZIP12* and *MTP2*.
**Figure S6.** Root DW of single and double Zn transporter mutant plants grown under Zn sufficiency and Zn deficiency.
**Figure S7.** Element concentrations of single and double Zn transporter mutant lines grown under Zn sufficiency or Zn deficiency.
**Figure S8.** Shoot to root ratio of element concentrations of *Arabidopsis thaliana* wild‐type, single and double Zn transporter mutant plants.
**Figure S9.** Normalized gene expressions of *Arabidopsis thaliana* single and double Zn transporter mutant plants grown under Zn deficiency.
**Figure S10.** Gateway destination vector constructed to clone the promoter sequences.
**Table S1.** T‐DNA insertion lines of Zn transporter genes with their respective primers for genotyping.
**Table S2.** Sequences of primers used for gene expression quantification by qRT‐PCR.
**Table S3.** Sequences of primers used to generate the destination vector.
**Table S4.** Sequences of primers used to clone gene promoters.

## Data Availability

Data available upon request from the authors.
