# Supplementary material for: Arabidopsis thaliana Zn transporter genes ZIP3 and ZIP5 provide the main Zn uptake route and act redundantly to face Zn deficiency
Source: Plant J. 2025 Feb 10;121(3):e17251. doi: 10.1111/tpj.17251 (PMC11811486; doi:10.1111/tpj.17251)
Supplement: Supplementary file 1 — Figure S1. Normalized gene expression in shoots and roots of Arabidopsis thaliana in response to Zn deficiency at increasing time points. Figure S2. Schematic drawings of Zn transporter genes with T‐DNA insertion. Figure S3. Confocal laser scanning microscope images of Zn distribution in Arabidopsis thaliana roots of wild‐type and Zn transporter mutant lines. Figure S4. Confocal laser scanning microscope images of Arabidopsis thaliana seedlings expressing nuclear‐localized YELLOW FLUORESCENT PROTEIN 2 (sYFP2) driven by the promoter regions of Zn transporter genes. Figure S5. Confocal laser scanning microscope images of Arabidopsis thaliana seedlings expressing nuclear‐localized YELLOW FLUORESCENT PROTEIN 2 (sYFP2) driven by the promoter regions of Zn transporters ZIP9, ZIP12 and MTP2. Figure S6. Root DW of single and double Zn transporter mutant plants grown under Zn sufficiency and Zn deficiency. Figure S7. Element concentrations of single and double Zn transporter mutant lines grown under Zn sufficiency or Zn deficiency. Figure S8. Shoot to root ratio of element concentrations of Arabidopsis thaliana wild‐type, single and double Zn transporter mutant plants. Figure S9. Normalized gene expressions of Arabidopsis thaliana single and double Zn transporter mutant plants grown under Zn deficiency. Figure S10. Gateway destination vector constructed to clone the promoter sequences. Table S1. T‐DNA insertion lines of Zn transporter genes with their respective primers for genotyping. Table S2. Sequences of primers used for gene expression quantification by qRT‐PCR. Table S3. Sequences of primers used to generate the destination vector. Table S4. Sequences of primers used to clone gene promoters. [file TPJ-121-0-s001.docx]

**Supporting Information**

## Article title: *Arabidopsis thaliana* Zn transporters complement each other to face Zn deficiency

Authors: Valeria Ochoa Tufiño, Maria Almira Casellas, Aron van Duynhoven, Paulina Flis, David E. Salt, Henk Schat, and Mark G.M. Aarts

The following Supporting Information is available for this article:

**Fig. S1.** Normalized gene expression in shoots and roots of *A. thaliana* in response to Zn deficiency at increasing time points.

**Fig S2.** Schematic drawings of Zn transporter genes with T-DNA insertion.

**Fig S3**. Confocal laser scanning microscope images of Zn distribution in *A. thaliana* roots of wild-type and Zn transporter mutant lines.

**Fig S4.** Confocal laser scanning microscope images of *A. thaliana* seedlings expressing nuclear-localized YELLOW FLUORESCENT PROTEIN 2 (sYFP2) driven by the promoter regions of *ZIP1*, *ZIP3*, *ZIP5*, *ZIP11*, *IRT3*, *HMA2*, and *YSL3*.

**Fig S5.** Confocal laser scanning microscope images of *A. thaliana* seedlings expressing nuclear-localized YELLOW FLUORESCENT PROTEIN 2 (sYFP2) driven by the promoter regions of *ZIP9*, *ZIP12*, and *MTP2*.

**Fig S6.** Root DW of single and double Zn transporter mutant plants grown under Zn sufficiency and Zn deficiency.

**Fig S7.** Element concentrations of single and double Zn transporter mutant lines grown under Zn sufficiency or Zn deficiency.

**Fig S8.** Shoot to root ratio of element concentrations of *A. thaliana* wild-type, single and double Zn transporter mutant plants.

**Fig S9.** Normalized gene expressions of *A. thaliana* single and double Zn transporter mutant plants grown under Zn deficiency.

**Fig S10.** Gateway destination vector constructed to clone the promoter sequences.

**Table S1.** T-DNA insertion lines of Zn transporter genes with their respective primers for genotyping.

**Table S2.** Sequences of primers used for gene expression quantification by qRT-PCR.

**Table S3.** Sequences of primers used to generate the destination vector.

**Table S4.** Sequences of primers used to clone gene promoters and promoters size.

**Fig S1.** Normalized Zn transporter gene expression in shoots and roots of *A. thaliana* in response to Zn deficiency at different time points. Gene expression relative to At5g25760 and At2g28390 in shoots and roots of plants grown for 15 days in fully supplied medium and then in either Zn sufficiency (full lines) or Zn deficiency (doted lines) during 108 hours. Samples were taken at increasing time points from 0.25 hours to 108 hours after applying the treatment. Average normalized expressions ± SE are shown on a ^10^log-scale, n = 3 samples of 3 pooled plants each.


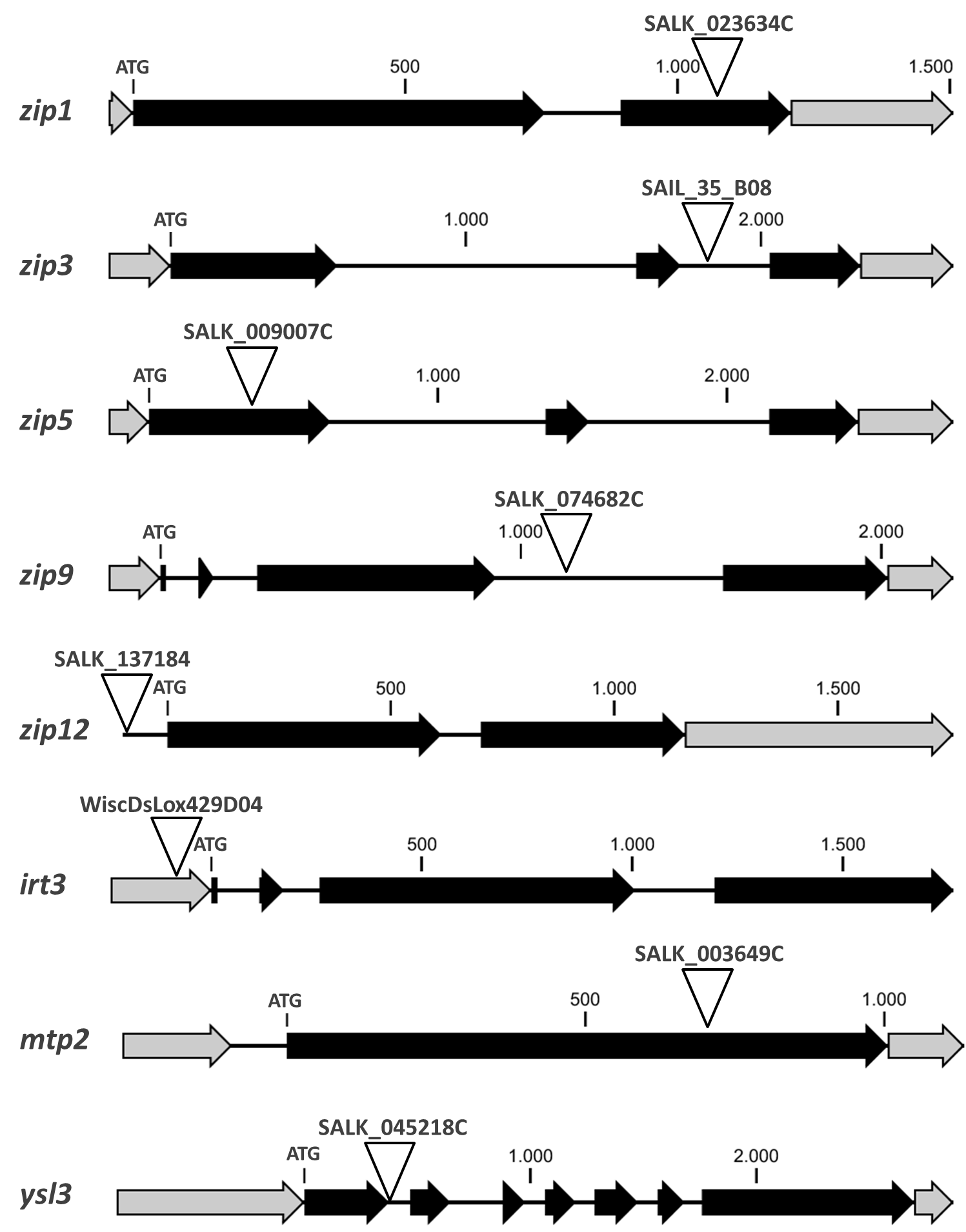


**Fig S2.** Schematic drawings of Zn transporter genes with T-DNA insertion. Gene sequences illustrating exons (black arrows), UTRs (grey arrows), untranscribed regions (including introns; black line), and the positions of the T-DNA insertions (white triangle) in mutant lines. Distance in base pairs to the start codon (ATG) is indicated above each gene.

**
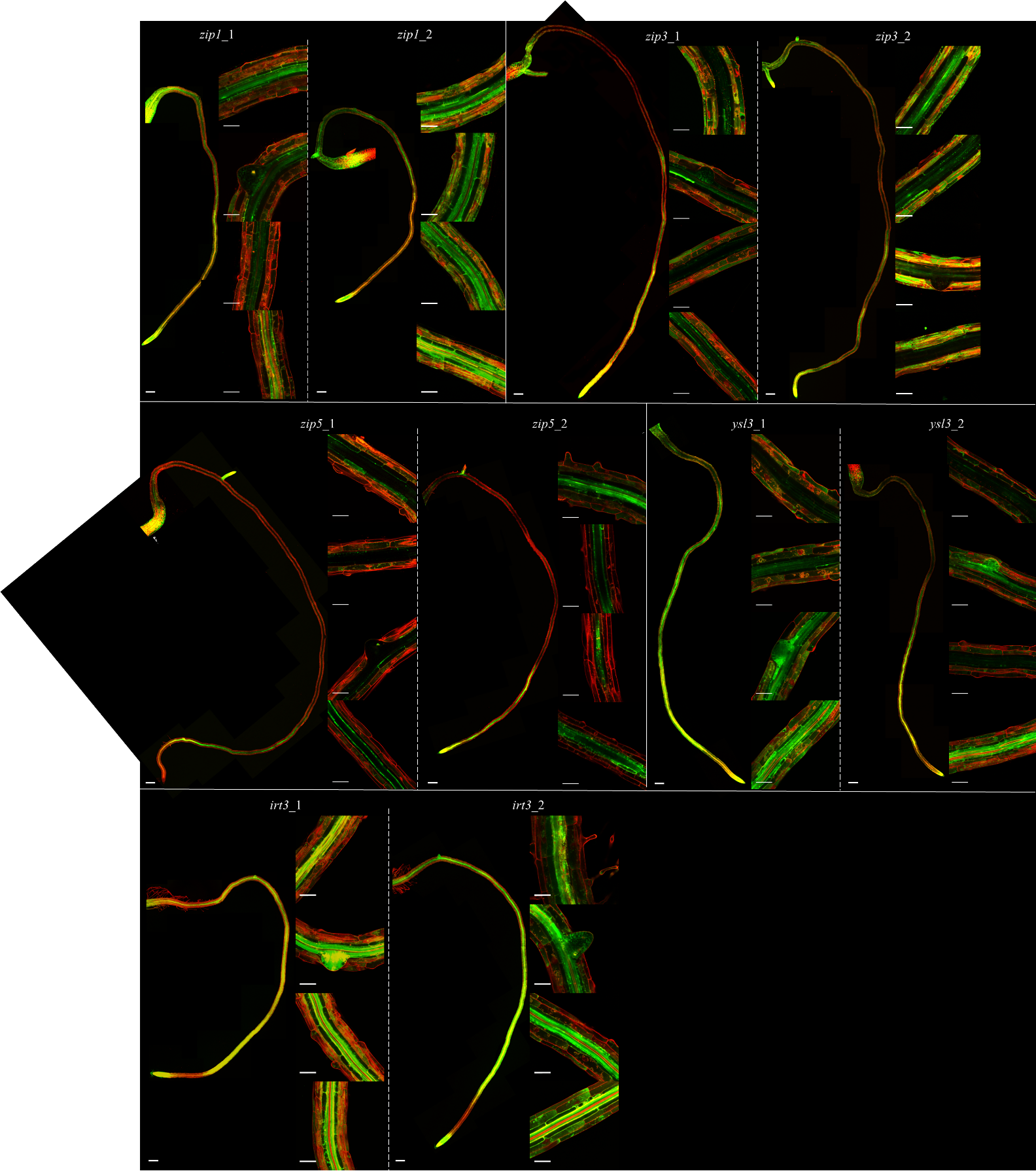
**

**Fig S3.** Confocal laser scanning microscope images of Zn distribution in *A. thaliana* roots of Zn transporters mutant lines. Zn deficient *A. thaliana* roots of five-day-old *zip1*, *zip3*, *zip5*, *ysl3* and *irt3* mutant plants incubated with Zinpyr-1 to detect Zn. Zinpyr-1-Zn complexes fluoresce green, propidium iodide bound to cell wall fluoresces red. Two plants and four of its sections are presented per mutant. The scale bars in full roots indicate 200 µm and in root sections indicate 50 µm


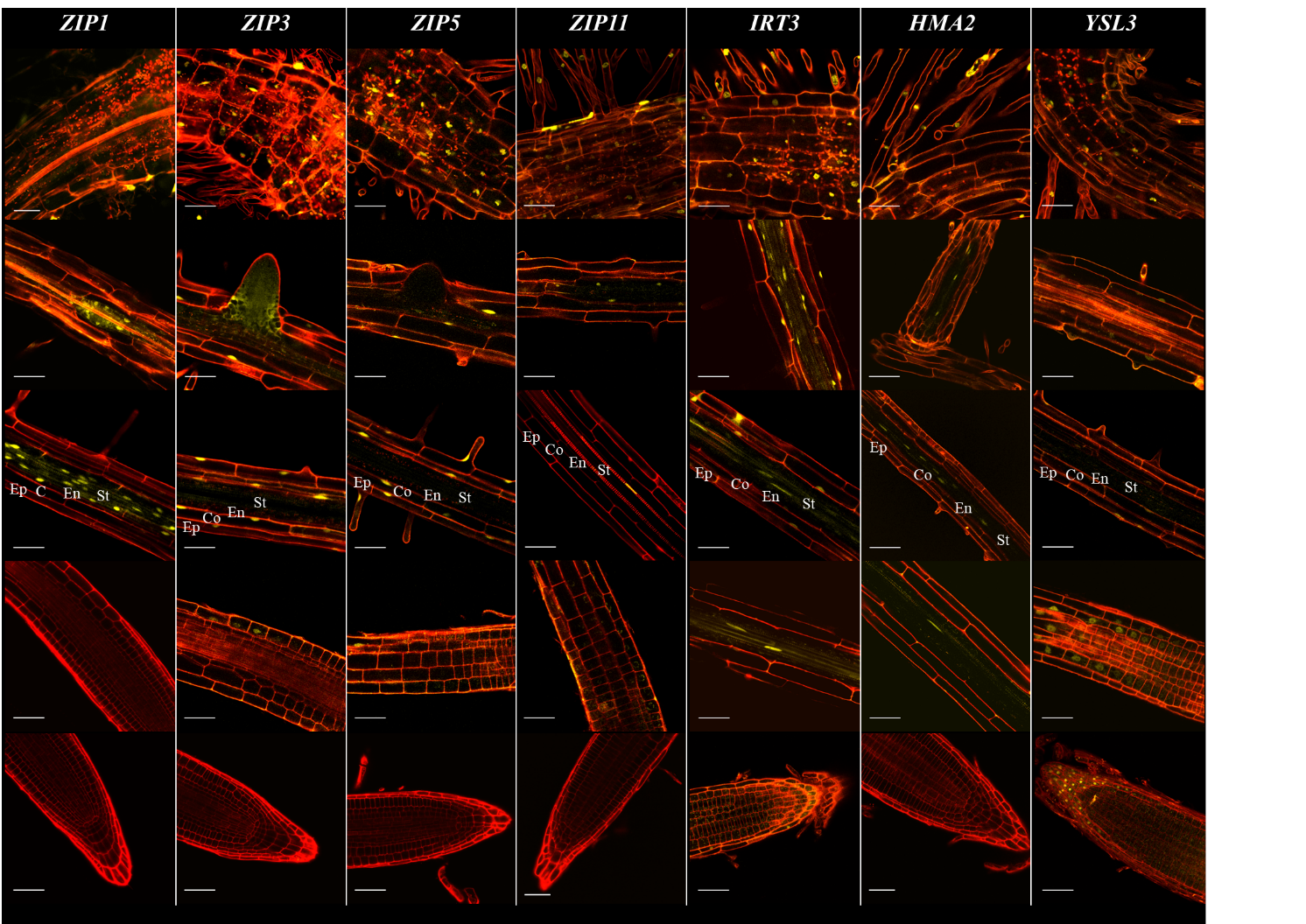


**Fig S4**. Confocal laser scanning microscope images of transgenic *A. thaliana* roots expressing nuclear-localised super YELLOW FLUORESCENT PROTEIN 2 (sYFP2) driven by the promoter regions of indicated Zn transporter genes. The YFP fluorescent signal (yellow) can be seen in nuclei of cells in which a promoter is active. Propidium iodide is used to indicate cell walls (red fluorescence). Four zones are distinguished, and detailed for each Zn transporter promoter-sYFP construct: the cell division (tip), elongation, differentiation and transition zones. In the middle panel of each column, a close-up of the differentiation zone is shown, in which the different cell layers of the root are distinguished: epidermis (Ep), cortex (C), endodermis (E) and stele (S) as indicated in the bottom panel of each column. Although the stele itself consists of different layers, the longitudinal images do not provide sufficient resolution to distinguish these. Occasionally larger yellow spots can be observed in the transition zone, exceeding the size of the nuclei, which corresponds to autofluorescent seed coat fragments. The scale bars indicate 500 µm in the whole root and 50 µm in the close-up images.

**
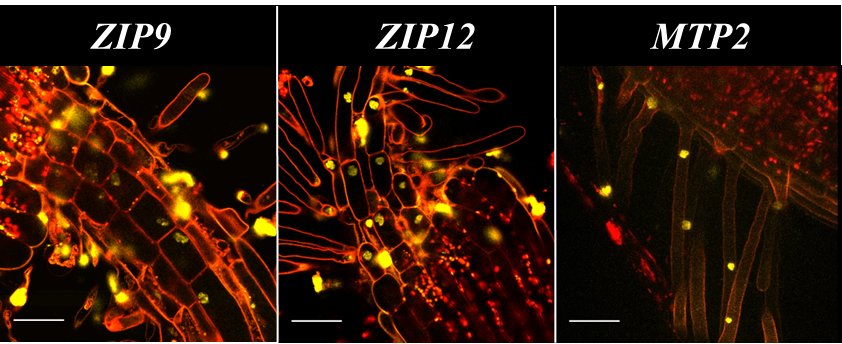
**

**Fig S5**. Confocal laser scanning microscope images of *A. thaliana* seedlings expressing nuclear-localized YELLOW FLUORESCENT PROTEIN 2 (sYFP2) driven by the promoter regions of Zn transporters *ZIP9*, *ZIP12*, and *MTP2*. The sYFP2 fluorescent signal (yellow) can be seen in the nuclei of cells expressing the NLS-sYFP2 marker gene. The red fluorescent signal indicates mainly cell walls stained with propidium iodide. These signals can only be seen in the *A. thaliana* root transition zone. Bright yellow spots larger than nuclei correspond to autofluorescence of seed fragments. The scale bars indicate 50 µm.

**
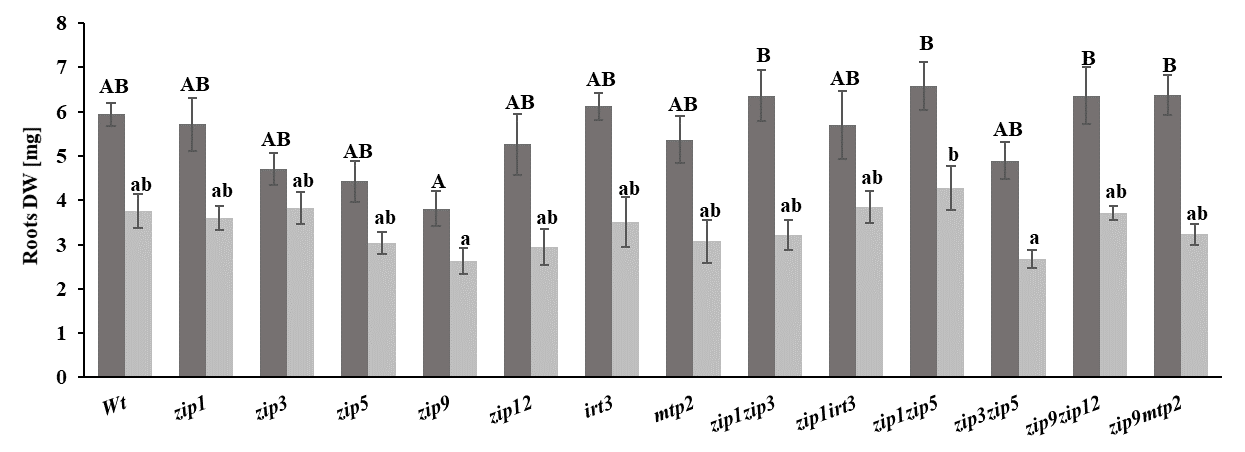
**

**Fig S6.** Root DW of single and double Zn transporter mutant lines grown under Zn sufficiency and Zn deficiency. Wild-type and single and double Zn transporter mutant lines grown hydroponically for 25 days under Zn sufficiency (dark bars) or for 10 days under Zn sufficiency and 15 days under Zn deficiency (light bars). Mean ± SE, n = 10 plants. Upper case letters (Zn sufficiency) and lower case letters (Zn deficiency) above the bars denote statistically different groups, when comparing among genotypes grown under each treatment, obtained with a Tukey post hoc test (α=0.05), after a one-way ANOVA (p<0.01).

**Fig S7.** Element concentrations of *A. thaliana* wild-type, single and double Zn transporter mutant plants grown under Zn sufficiency or Zn deficiency. (a) Shoot and (b) root Cu, Fe, K, Mg, Mn, Mo, Na, P, S, and Zn concentrations of plants grown hydroponically for 25 days under Zn sufficiency (dark bars) or for 10 days under Zn sufficiency and 15 days under Zn deficiency (light bars). Mean ± SE, n = 5 plants. Upper case letters (Zn sufficiency) and lower case letters (Zn deficiency) above the bars denote statistically different groups, when comparing among genotypes grown under each treatment, obtained with a Tukey post hoc test (α=0.05), after a one-way ANOVA (p<0.01).

**Fig S8.** Shoot to root ratio of element concentrations of *A. thaliana* wild-type, single and double Zn transporter mutant plants grown under Zn sufficiency or Zn deficiency. Ratio of Cu, Fe, K, Mg, Mn, Mo, Na, P, S, and Zn concentrations of plants grown hydroponically for 25 days under Zn sufficiency (dark bars) or for 10 days under Zn sufficiency and 15 days under Zn deficiency (light bars). Mean ± SE, n = 5 plants. Upper case letters (Zn sufficiency) and lower case letters (Zn deficiency) above the bars denote statistically different groups, when comparing among genotypes grown under each treatment, obtained with a Tukey post hoc test (α=0.05), after a one-way ANOVA (p<0.01).

**Fig S9.** Normalized gene expressions of *A. thaliana* wild-type and single and double Zn transporter mutant lines grown under Zn deficiency. Plants grown hydroponically for 10 days under Zn sufficiency and 12 days under Zn deficiency. Gene expression of shoots (dark bars) and roots (light bars) was normalized to the expression of two reference genes (At5g25760 and AT2G28390). Average normalized expressions ± SE are shown on a ^10^log-scale, n = 3 samples of 2 pooled plants each. Upper case letters (shoot) and lower case letters (root) above the bars denote statistically different groups, when comparing among genotypes grown under each treatment, obtained with a Tukey post hoc test (α=0.05), after a one-way ANOVA (p<0.01).


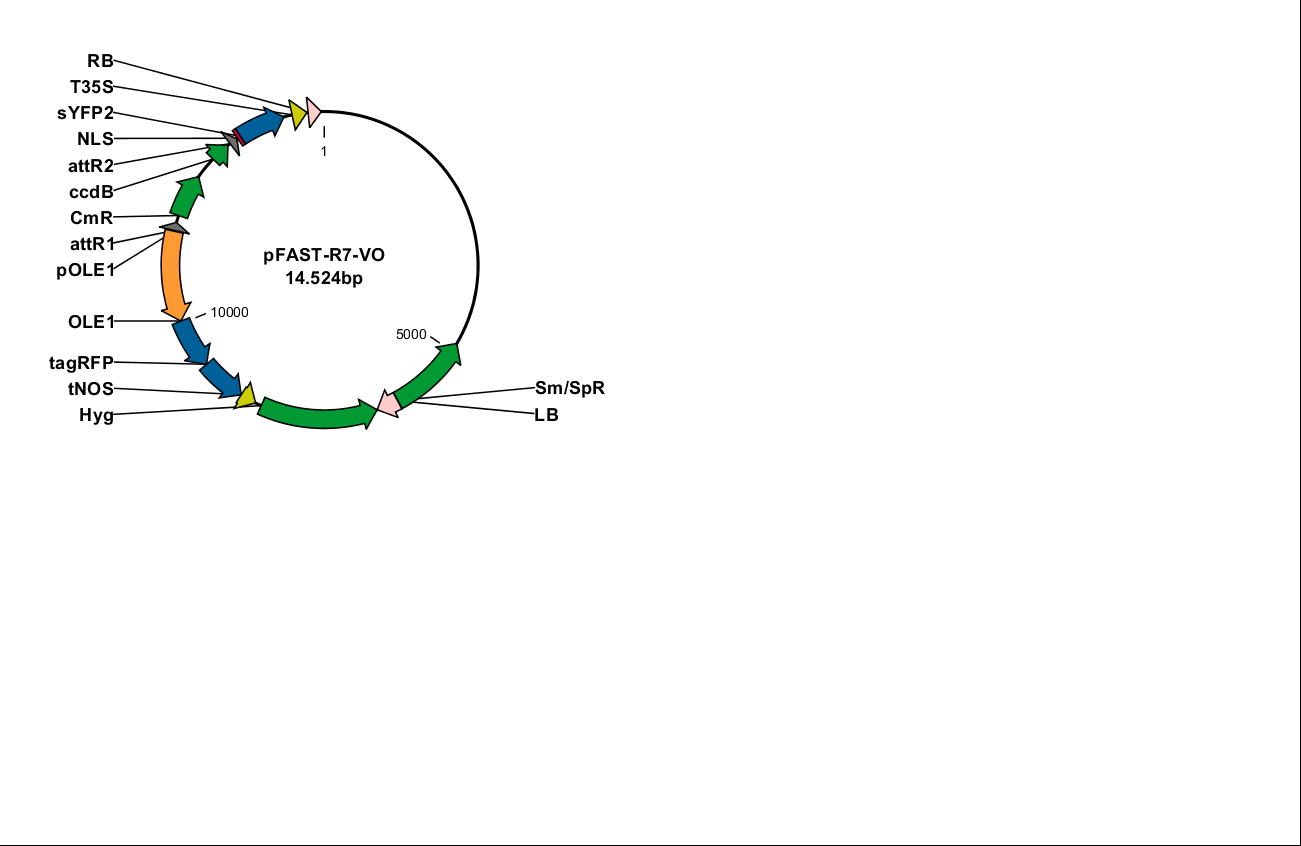


**Fig S10.** Gateway destination vector constructed to clone the promoter sequences, derived from the FAST-R07 vector plasmid (Shimada et al., 2010).

**Table S1.** T-DNA insertion lines of Zn transporter genes with their respective LB (left border) and RP (right border) primers for genotyping.

| **Symbol** | **Locus identifier** | **T-DNA line** | **Primer** | **Sequence (5’-3)** |
| --- | --- | --- | --- | --- |
| *ZIP1* | At3g12750 | SALK_023634C | LB | CTTGAGGACACTACAGCTGGG |
|  |  |  | RB | GAATGCGGATTATTAGCTGG |
| *ZIP3* | At2g32270 | SAIL_35_B08 | LB | GGCTCGTTACTTTCCTTGGAC |
|  |  |  | RB | CCATTATTTGGAATGCAATCG |
| *ZIP5* | At1g05300 | SALK_009007C | LB | AGGGACAAAAACAAATACGGG |
|  |  |  | RB | AGGCAAATGAAAATTCAGTGAC |
| *ZIP9* | At4g33020 | SALK_074682C | LB | ACACCGATCGGAGTAGTGATG |
|  |  |  | RB | TTTCGGTTATGTCTGACCCAG |
| *ZIP10* | At1g31260 | SALK_104586C | LB | TTTGCTTTTATTTGAGTGACAACC |
|  |  |  | RB | TCTGCATTCCATAAATGTCAAG |
| *ZIP11* | At1g55910 | SALK_120099C | LB | TTGGTTGATCTTCCTGTTTGG |
|  |  |  | RB | AGGATTTGGATTTGAGATCGG |
| *ZIP12* | At5g62160 | SALK_137184 | LB | AATGTCCTTGTGAGGCATGAG |
|  |  |  | RB | GATTTCTCCCTGGTCGAAGAG |
| *IRT3* | At1g60960 | WiscDsLox429D04 | LB | TGATGTGTGTTTTGCTCTTCG |
|  |  |  | RB | AGCAATGGATAAGAAATTGCG |
| *MTP2* | At3g61940 | SALK_003649C | LB | GCTGCAGATGGATTCAAGAAG |
|  |  |  | RB | ATGAGAGCATACGAGAAAGCG |
| *YSL3* | At5g53550 | SALK_045218C | LB | GCCTTTAGGAGTGTGGAAACC |
|  |  |  | RB | TTTTTCCTCTCGTCATTTTCC |
| *HMA2* | At4g30110 | SALK_034393 | LB | GGAGAGTGACTCCCTAAAGCC |
|  |  |  | RB | TAAACAAGAATGGCGTCGAAG |

**Table S2.** Sequences of primers used for gene expression quantification by qRT-PCR.

| **Gene** | **Primer Forward** | **Primer Reverse** |
| --- | --- | --- |
| *ZIP1* | TCTCCCTGGCGGATATGAAGTC | TCCACCATTATTGCCTCTTTGCTC |
| *ZIP3* | CTCCTTCTCATCGCCGTCGT | CGAGCTCCGGCTTTGTTTTC |
| *ZIP4* | GGCTGCATCTCTCAGGCACA | GGCCACTGCAGTTCCAATCC |
| *ZIP5* | CGTCGCTTAAACCGGAGACG | CATAAACCCCGTTGCGAGGA |
| *ZIP9* | ACTTGTGTACATGGCGCTTG | TGCAAGAGCAGACATCATCC |
| *ZIP11* | TTGGTACACAATTCGCCGGA | AAGCAAATGGGTAAGCCGGA |
| *ZIP12* | CCATCTTAATCGCCGGAGTA | TCTTCTCCAAGGCACGAACT |
| *IRT3* | GATTCTCGCCACGGGTTTTG | GCAAAGAATCCGGGAAAGG |
| *HMA2* | TCCGTCAAGAACCGTCATCGTC | AACGATTTGGAACTGCGAGAGG |
| *MTP2* | CGAGCTGCTTCAATGCGAAA | AGCACCAACATCAGTGAGCA |
| *YSL3* | GGTGGTACAGAGTGCGGTTT | TGATTCCCCAAGACAGAACC |
| *SAND* | GTTGGGTCACACCAGATTTTG | GCTCCTTGCAAGAACACTTCA |
| *PEX4* | TCCTGAGCCGGACAGTCCTC | CATAGCGGCGAGGCGTGTAT |

**Table S3.** Sequences of primers used to generate the destination vector. Underlined bases represent the primer linkers. NLS = nuclear localization signal; sYFP = super YELLOW FLUORESCENT PROTEIN; DJ = double joint.

| **Primers description** | **Sequence (5'- 3')** |
| --- | --- |
| Forward for NLS | ATTCACATTCTTGCCCGCCT |
| Reverse for NLS plus a sYFP2 linker | GCCCTTGCTCACCATAGGTTGAGAAGATGGCTCTATTTTC |
| Forward for sYFP2 plus a NLS linker | CCATCTTCTCAACCTATGGTGAGCAAGGGCGAGGAGCTGT |
| Reverse for sYFP2 | CCTTCAACGTTGCGGTTCTG |
| Forward for the DJ fragment | TCTTGCCCGCCTGATGAATG |
| Reverse for DJ fragment plus a *Nru*I linker | TTCTCGCGATCTAGTAACATAGATGACAC |

**Table S4.** Sequences of primers used to clone gene promoters (Prom.). Forward primers include the Gateway attB1 linker (GGGGACAAGTTTGTACAAAAAAGCAGGCTTA) and reverse primers include the Gateway attB2 linker (GGGGACCACTTTGTACAAGAAAGCTGGGTA) at their 5’ ends.

| **Promoter** | **Primer Forward** | **Primer Reverse** | **Fragment Size (bp)** |
| --- | --- | --- | --- |
| *ZIP1* | CAGTTATGCAAATACTCCGG | TGAGTTTAAGATATTTATGTTCTTGTT | 1542 |
| *ZIP3* | TATCCAGAAAGATGATGTGTATACA | AATCTCTATCTTATTTTAAAATTAGGG | 1798 |
| *ZIP5* | TATGTTTTTATTTTGAGGCACAAATT | CTTATCGATTAGGGTTTGAATTTGA | 1798 |
| *ZIP9* | CAGATATGCCCAAAGCATATTCTT | TAGCTGCGAACTTGAGGGTAA | 1142 |
| *ZIP10* | TTGTCTTCTTTTTCCCGCTTC | CTTTCTATTTGTTTCTTGTGGAGTTTT | 981 |
| *ZIP11* | GAACTTTGAGAAATTATTGGTTGAAAC | TTTGAATGTTCAGTGGGTTTGT | 426 |
| *ZIP12* | GAGTTGTGTACCGTGAGTTGATTATT | TCGTTTACTTTTGACAAAAGTTAGG | 1325 |
| *IRT3* | ACTCTTTTTCATGTTCCATACACAATT | TTGGGGTCTAAGATGTCCTCG | 787 |
| *MTP2* | CATCTCAAAAGGTAAACTTAAAGACAA | CTGCAGCAAAAAAGATTGTAACTTT | 1244 |
| *HMA2* | AACTATTCCAAATGAGGAATCACAC | TCTTGTTTAAGGATTCTGCA | 1754 |
| *YSL3* | CGTCCGAATATAATAGTCCAATTCTAC | TTTTTTCCAAGAACAGAACAAAAAA | 1801 |
